# Supplementary material for: Palaeodemographic modelling supports a population bottleneck during the Pleistocene-Holocene transition in Iberia
Source: Nat Commun. 2019 Apr 23;10:1872. doi: 10.1038/s41467-019-09833-3 (PMC6478856; doi:10.1038/s41467-019-09833-3)
Supplement: Supplementary file 1 — Supplementary Information [file 41467_2019_9833_MOESM1_ESM.pdf]

Supplementary Information

Palaeodemographic modelling supports a population bottleneck during the Pleistocene-Holocene transition in Iberia

Javier Fernández-López de Pablo* [1,2], Mario Gutiérrez-Roig[3], Madalena Gómez-Puche[1,2], Rowan McLaughlin[1,2], Fabio Silva[4], Sergi Lozano[1,2]

*Corresponding author: jfernandez@iphes.cat

1 Institut Català de Paleoecología Humana i Evolució Social (IPHES), Edificio W3, Campus Sescelades URV, Zona Educacional 4, 43007 Tarragona, Spain

2 Àrea de Prehistòria, Universitat Rovira i Virgili, Avda. Catalunya 35, 43007, Tarragona Spain.

3 Behavioural Science Group, Warwick Bussiness School, University of Warwick, Scarman Rd, Coventry CV4 7AL, United Kingdom

4 Centre for Archaeology and Anthropology, Faculty of Science & Technology, Bournemouth University, Fern Barrow, Poole, Dorset, BH12 5BB, United Kingdom

SUPPLEMENTARY INFORMATION

Supplementary Methods, 1

1. PALEODEM radiocarbon database description, 1
2. Data quality control and filtering criteria, 3
3. Binning process, 6
4. Calibration procedures, 6
5. Temporal frequency distribution of radiocarbon dates and archaeological sites,9
6. Chronological modelling: Summed Probability Distributions and bootstrapping, 10
   6.1 Taphonomic correction and non-linears caling adjustment, 12
   6.2 Bootstrapping confidence intervals, 13
   6.3 Required number of simulation runs, 14
   6.4 Effects of the calibration curve(atmospheric or marine)on the observed SPD, 16
   6.5. Choice of calibration curves and back-calibration datasets for simulation, 18
   6.6. Testing a null model, 19

7.Model Selection and Demographic Growth Rates, 20
   7.1. Motivation and Choice of Demographic Model(s), 20
   7.2. Model Selection Methodology, 21
   7.3. Results, 22
   7.4. Annual Growth Rates and Associated Uncertainty, 23

Supplementary Discussion, 25

8.Regional population patterns,25
   8.1. Cantabrian subset Ebro Valley subset, 27
   8.3. Mediterranean subset, 28
   8.4. Atlantic subset, 29
   8.5. Interior subset, 30

9.Correlation with proxy records of palaeoenvironmental change, 30

Supplementary References, 34

Supplementary Information

The following supporting information provides: i) the radiocarbon database analyzed in this study along further details about the data quality control, binning and calibration procedures; ii) a description of the methodological steps followed in the radiocarbon palaeodemographic modelling, including the links to a GitHub repository where the *ad hoc* Python and R scripts used in the statistical analyses and visualization are freely available; iii) additional discussion about regional population patterns based on regional SPDs analyses; and iv) additional information about the statistical methods used for model selection and the calculation of demographic growth rates.

Supplementary Methods

1. PALEODEM radiocarbon database description

A new georeferenced database of radiocarbon dates (hereafter PALEODEM radiocarbon database) has been created for this study using bibliographic references. It includes 1198 ^{14}C determinations, from 795 assemblages in 246 archaeological sites, covering the period between the Early Magdalenian and the end of the Late Mesolithic in the Iberian Peninsula. This dataset encompasses the most up-to-date list of archaeological ^{14}C dates for this time range in the Iberian Peninsula. The complete list of sites, their descriptions and the corresponding bibliographic references are available on Zenodo – doi: 10.5281/zenodo.1145698.

We have taken an all-inclusive approach during early stages of the data collection process, considering the total number of radiocarbon determinations, under the premise that large samples would built a more representative dataset. However, there is an undoubtedly need for critically auditing the database, as discussed below.

Potential methodological and archaeological problems could affect the accuracy of a radiocarbon determination. These issues are related with sample pretreatment techniques, the method of radiocarbon measurement (whether conventional or AMS), as well as constraints concomitant with the characteristics of the sample itself and its associated archaeological context. Supplementary Table 1 shows the absolute and relative frequencies of radiocarbon dates according to the sample material and their rank of chronological precision, itself based on the inbuilt age of each material (1-3).

Charcoal is the most common dated sample in the Iberian record (44% of the total), especially detrital charcoal found dispersed into stratigraphic layers. This sample type might be affected by the well-known and extensively discussed “old wood effect” (2, 4). Dating short-lived samples taxonomically identified reduces the uncertainty of the inbuilt

age of radiocarbon dates. We have distinguished between short-lived (n=53) and long-lived charcoal samples (n=481), when that information was available in the literature.

Sample	Inbuilt sample age	Rank	n	%
Seeds fruits	0-1	1	11	1
Short lived charcoal	0-1	1	53	4
Human bone (tooth inl.)	<20	1	79	7
Bone	<20	1	358	30
Long lived charcoal	<100	2	481	40
Marine shells	>100	3	150	13
Pollen	Unknown	4	1	0
Sediment	Unknown	4	14	1
Organic paint	Unknown	4	12	1
Land snail shells	Unknown	4	2	0

Supplementary Table 1. Absolute and relative frequencies of different sample types from the PALEODEM project database (period 18,000-7,500 cal BP).

Bone samples are the second largest material of the PALEODEM database (n=358). When the information was available, we have differentiated between individual samples (n= 131), and aggregated samples (n=63), since that trait has direct implications for the degree of reliability of the carbon measurement in the sample (1). At present, bone is considered one of the most reliable materials to date, whether the collagen fraction or the structural-mineral fraction is concerned. The reliability of dating cremated bones has also been demonstrated (5-6). However, anomalously low $\delta^{13}C$ values have been reported for calcined bones, suggesting that the mineral bone fraction is somehow altered, and exchange with other sources of carbon during heating cannot be excluded (7). In this regard, the assessment of the ratio C:N has been established as a quality indicator for radiocarbon dating of bone samples (8).

Despite the advances on removing carbon contaminants from archaeological bone samples (e.g. by means of ultrafiltration protocol), recent studies have reported how the amount of residual ^{14}C in the bone can be significantly altered by the inclusion of any small fraction of unknown carbon source, producing large laboratory errors (2, 3, 9). This problem particularly affects samples dated beyond ~2 or ~3 half-lives (i.e. dates > 11460 BP) where residual ^{14}C is already low. Nevertheless, as far as the PALEODEM database is concerned, more than 87% of radiocarbon dates are outside the problematic time range.

Marine shell samples represent 13% of the radiocarbon dates in PALEODEM database. These cases are affected by the “marine reservoir effect” (MRE) (10-12). The

carbon accumulated in marine shells comes from a mixing of different sources, which cause a deviation from atmospheric carbon. Moreover, the variation in the marine reservoir effect, which depends on whether the marine mollusk species are filter feeders (e.g. *Cerastoderma* or *Scrobicularia* genus) or grazers (e.g. *Mytilus* genus), should be taken into account (3, 13). MRE also exists in samples from human remains where there is evidence of a significant marine dietary intake (these cases represent 4% of the PALEODEM database). Ultimately, although the number of radiocarbon dates affected by MRE in the PALEODEM database represents a low percentage of the whole assemblage (14%), it is necessary to consider the ΔR value and calibrate dates with the appropriate marine/mixed curve to obtain more precise and reliable chronometric measurements, particularly in the Early Holocene section.

Pollen, bulk sediment, organic pigment or land snails samples are minimally represented in the PALEODEM database. This kind of samples are problematic for obtaining accurate radiocarbon ages given the possible combination of different sources of carbon. The most evident example is the “limestone effect” recognized on land snail samples (14, 15).

2. Data quality control and filtering criteria

Similarly to other works analyzing large radiocarbon datasets (16, 17), we have filtered the PALEODEM database to identify potentially unreliable ^{14}C dates. The following tables summarize the set of filtering criteria applied to accept the validity of the radiocarbon ages analyzed in this study according to the sample (Supplementary Table 2), and its association with the archaeological context (Supplementary Table 3), respectively.

Accepted ^{14}C dates	Rejected ^{14}C dates
Individual seeds and fruits, taxonomically identified<br><br>Individual short-lived charcoal<br><br>Individual bones<br><br>Individual long-lived charcoal<br>Marine shells and human remains (with known ΔR)	Pollen<br><br>Bulked sediment<br><br>Land snail shells<br><br>Charcoal and organic pigments
Samples with SD ≤ 200	Samples with SD > 200

Supplementary Table 2. Filtering criteria applied concerning the sample type and the laboratory error.

Accepted ^{14}C dates	Rejected ^{14}C dates
Individual short-lived charcoal, spatially associated with anthropogenic features<br><br>Individual/aggregated long-lived charcoal of detrital origin, when consistent with the chrono-cultural interpretation<br><br>Aggregated bone, when dates are consistent with the chrono-cultural interpretation	Bulk charcoal and bulk bones samples, when inconsistent with relative cultural attribution
Reliable samples in undisturbed archaeological contexts	Reliable samples in severely disturbed archaeological contexts

Supplementary Table 3. Filtering criteria applied concerning the uncertainty of association and stratigraphic interpretation.

We have retained for analysis all the individual radiocarbon ages measured on taxonomically identified samples of palaeobotanical remains (seeds and fruits), as well as short-lived charcoals identified to genus level, both stratigraphically associated with, and spatially related to, anthropic contexts. In addition, samples from individual bones and individual long-lived charcoal have been accepted if there was a high probability of association with anthropic activity (e.g. hearth, fireplace, burial, …).

Additionally, we have admitted those radiocarbon dates from individual samples of marine shell species with known ΔR value and from aggregated samples of bone and charcoal, if the ^{14}C determinations were consistent with the chrono-cultural interpretation of the site and the archaeological sequence based on the artifactual evidence.

A filter for laboratory errors of uncalibrated ages equal to, or lower than 200 years, regardless of the method of radiocarbon measurement - conventional or AMS - was also established in order to accept radiocarbon ages for further statistical analysis, thereby rejecting those dates that have reported higher error values (n=127). In addition, samples of bulk sediment or pollen (n=15) with no clear association with anthropogenic occupational activity were also discarded. Finally, land snails, marine shells with an unknown reservoir effect, and samples of organic pigments, were also discarded (n=60). By applying the filtering criteria specified above, we retained 907 radiocarbon dates and excluded 291.

An exploratory error analysis of the standard deviation indicates that over 90% of the dates have errors of 200 years or less, and 67% have errors of 100 years or less (Supplementary Figure 1). In sum, the mean of laboratory errors for the entire PALEODEM database is 118 years and 115 years for the filtered subset.

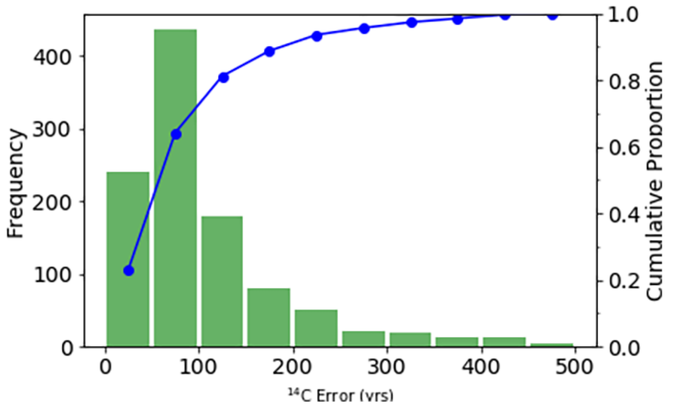

Supplementary Figure 1. Histogram (50-year bins) of ^{14}C laboratory errors of the radiocarbon dates in PALEODEM database. Green bars represent the absolute frequency and blue line their cumulative proportion (following 18).

The correlation between filtered and unfiltered radiocarbon dates in the PALEODEM database shows a modest correlation coefficient (r=0.48), demonstrating that the results of any analysis carried out in them will be different, depending on whether one uses one subset or the other (Supplementary Figure 2).

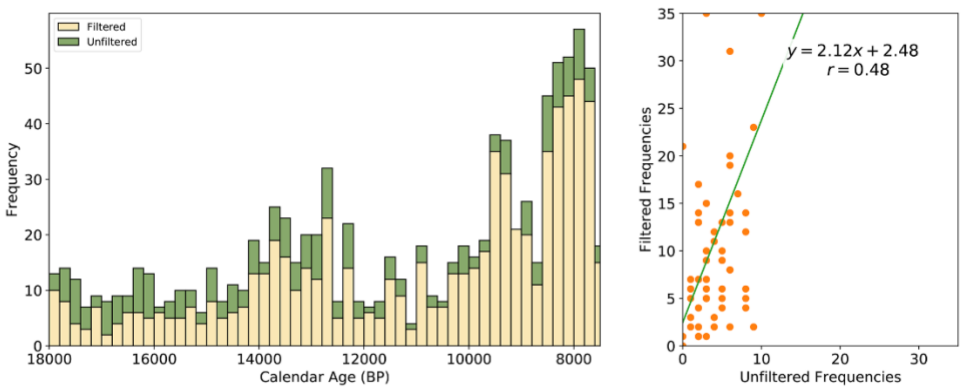

Supplementary Figure 2. Frequency of filtered and unfiltered radiocarbon dates in the PALEODEM database. Left: Histogram of 200-year bins based on the median of the calibrated range for each radiocarbon date. Yellow bars show the frequency distribution of the filtered radiocarbon dates, whereas green bars show the unfiltered frequencies. Right: Scatter-plot with filtered and unfiltered radiocarbon dates and the result of a linear regression analysis.

3. Binning process

Intra-site sampling bias or "wealth bias" may affect the representativeness of different archaeological phases and blur their weight in the reconstruction of palaeodemographic dynamics from chronological time-series analysis (18, 19). To mitigate the over-representation of radiocarbon dates from the same archaeological layer or context, we have followed a semi-manual process for combining radiocarbon dates within 200-year bins, prior to calibration. We set the bin widths at 200 yrs because the mean of the laboratory errors in the study (the filtered dataset used for analysis) is 115 ^{14}C years. Therefore, 200 years has a suitable heuristic value for identifying and pooling dates that may derive from the same phase of activity, without mistakenly ignoring phases of activity that are separate in time, which may be a more serious error in regards to the results. We have used the *R_Combine* and *Combine* functions of Oxcal software, according to the sample material of each radiocarbon date, and following the instructions of the most recent version of the Oxcal 4.3 Manual (http://c14.arch.ox.ac.uk/oxcalhelp/hlp_contents.html). These functions perform a chi-squared test, the result of which is used to determine whether two or more radiocarbon dates from the same source or context are statistically equivalent (20) and, consequently, if they can be combined within the same bin. After this process, the pruned database included 783 radiocarbon dates for calibrating.

4. Calibration procedures

Calibration routines have been implemented in IOSACAL, an open-source Python package (http://iosacal.readthedocs.io/en/latest/). The corresponding script is available on GitHub (*calibrate_single_date_mixed_curve.py* in repository *PALEODEM/Palaeo-demographic-models*).

All radiocarbon dates have been calibrated using the Intcal13 and the Marine13 calibration curves (21). Those samples requiring marine calibration or mixed marine-atmospheric calibration (marine shell samples and human bones with marine diet intake), have been calibrated with an especially developed "mixed calibration and reservoir effect" function allowing to mix two different calibration curves in a certain proportion, and to introduce the local ΔR correction value. We have applied the values defined in the original publications when the researchers have provided them (Supplementary Table 4 and Supplementary Figure 3). In the case of the Mediterranean coast, we have used the online marine reservoir correction database accessed at the website http://calib.org (22), where the ΔR value represents the weighted mean for this geographic region and is expressed in ^{14}C years. Additionally, radiocarbon dates from the Cantabrian region will need different ΔR according to their specific chronologies (12). Ultimately, we have implemented this particular function to obtain more precise and reliable absolute chronologies in our study, however, there is still a lively debate about the methods used in determining regional differences in marine radiocarbon reservoir effect (23, 24).

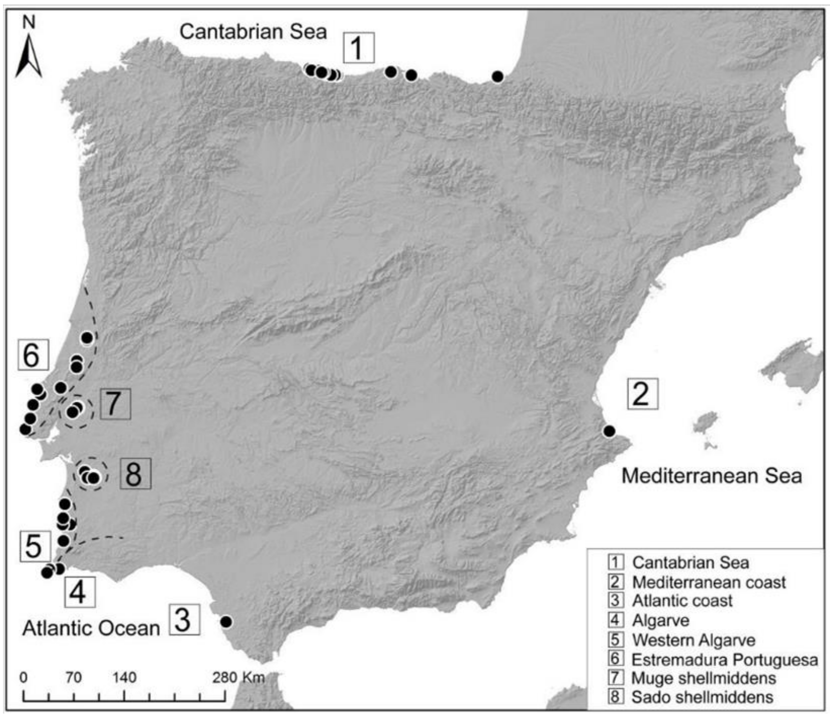

Supplementary Figure 3. Geographic distribution of archaeological sites with radiocarbon dates subject to marine calibration. The numbers refer to the different local ΔR values according to specific geographic locations considered in this study and referred in Supplementary Table 4. The background map has been generated in ArcGIS 10.3 using 1 arc-second SRTM raster data (available from https://earthdata.nasa.gov/) to represent terrain via a hillshade model.

ΔR value	References	Region	Site ID
69 ± 17	Monge-Soares et al., 2016	Algarve	2
-110 ± 15	Valente et al., 2014	Western Algarve	166
-116 ± 40	Valente et al., 2014	Western Algarve	168
-116 ± 44	Valente et al., 2014	Western Algarve	145
380 ± 30	Bicho et al., 2010	Estremadura portuguesa	4, 146
95 ± 15	Carvalho 2010	Estremadura portuguesa, Alentejo	11, 134, 140, 141, 143, 144, 147, 150, 151, 163, 164, 165, 167, 248, 249
140 ± 40	Martins et al., 2008	Muge shellmiddens	127, 128, 129, 132
-150 ± 155	Araujo 2015	Sado shellmiddens	130, 131, 133, 158, 159, 160
-105 ± 21	Monge-Soares et al., 2016	Cantabrian Sea - Holocene	80, 85, 98, 108, 110, 111, 114, 115, 117, 119, 120, 122, 237
-117 ± 70	Monge-Soares et al., 2016	Cantabrian Sea - Pleistocene	87
95 ± 61	Gibaja et al., 2015	Mediterranean coast of Spain	124
-108 ± 31	Monge-Soares et al., 2016	Atlantic coast of Spain	242

Supplementary Table 4. Local ΔR values specifically applied to each site of the PALEODEM database.

5. Temporal frequency distribution of radiocarbon dates and archaeological sites

After the calibration process, the medians of the calibrated ages have been aggregated in two different frequency histograms: the first one showing dates in 200-year bins (Supplementary Figure 4, top), and the second showing archaeological sites by millennia (Supplementary Figure 4, bottom). In both histograms, the frequency distributions are represented according to three different categories of archaeological sites – caves, rock shelter, and open-air deposits – which allows one to assess, at the exploratory level, the potential effects of taphonomic bias and settlement patterns. The scripts for both frequency histograms are available on GitHub (*frequencies_histogram.py* and *frequencies_histogram_type_sites.py* in repository *PALEODEM/Palaeo-demographic-models*).

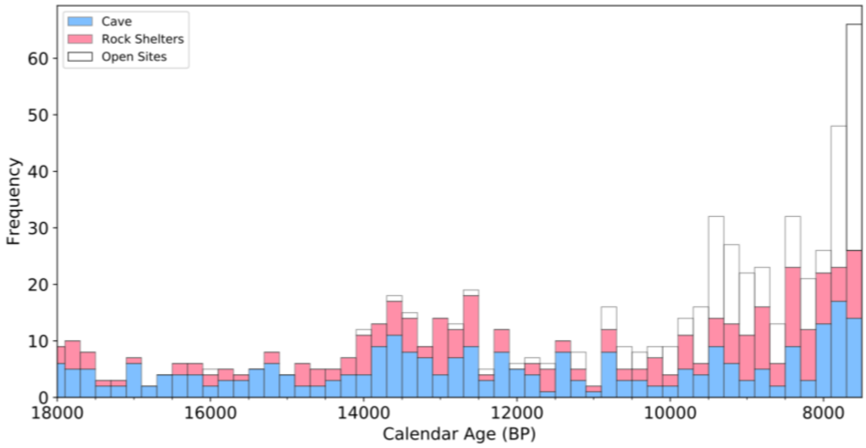

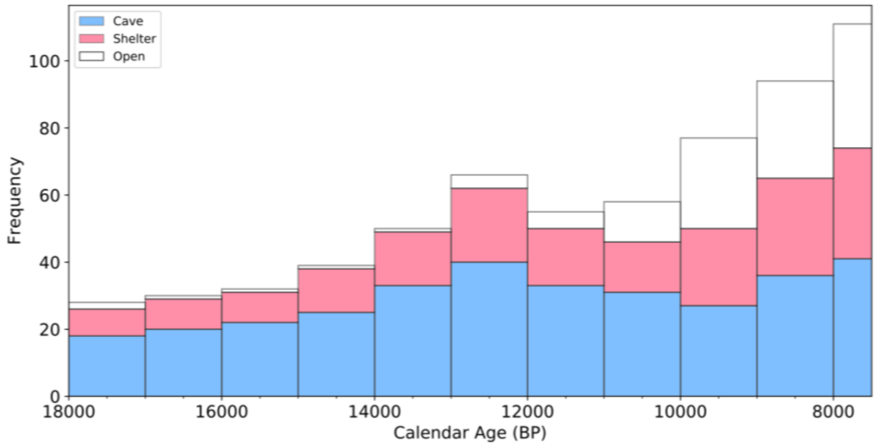

Supplementary Figure 4. Frequency distribution of radiocarbon dates and sites. Top. Histogram (200-year bins) of calibrated filtered dates' medians. Bottom. Frequency distribution of archaeological sites by millennia.

The frequency distribution of calibrated radiocarbon dates by site type (Supplementary Figure 4, top) clearly shows a dominance of radiocarbon dates from closed sites (whether caves or rock shelter archaeological deposits) along the Late Glacial section of the analyzed time series (18-13 kya). This phenomenon is consistent with the variation in number of sites by millennia (Supplementary Figure 4, bottom), in both cases due to field-oriented research biases. In contrast, from the Early Holocene onwards we observe a monotonic increase in both the number of radiocarbon dates from open-air sites and in the number of open-air sites by millennia. This pattern of frequency distribution on the open-air archaeological record fits well with the so-called "taphonomic bias", a time-dependent destruction of sites and datable material (25).

6. Chronological modelling: Summed Probability Distributions and bootstrapping

In this study, we have used a chronological modelling approach based on the analysis of Summed Probability Densities (SPD) of archaeological radiocarbon dates. Supplementary Figure 5 illustrates the sequence of work processes and analytical procedures followed to obtain the population proxy. The procedures regarding the database collection, error analysis, filtering, binning processes and radiocarbon calibration have been previously described (Supplementary Sections 1 and 4). Once the ^{14}C dates were calibrated, we have followed two different work-processes. On one hand, we calculated the medians of each single radiocarbon date and aggregated them in frequency histograms of dates and sites (as detailed in Supplementary Section 5); whereas on the other hand, the normalized probability distribution of all individually-calibrated dates were aggregated to produce a SPD.

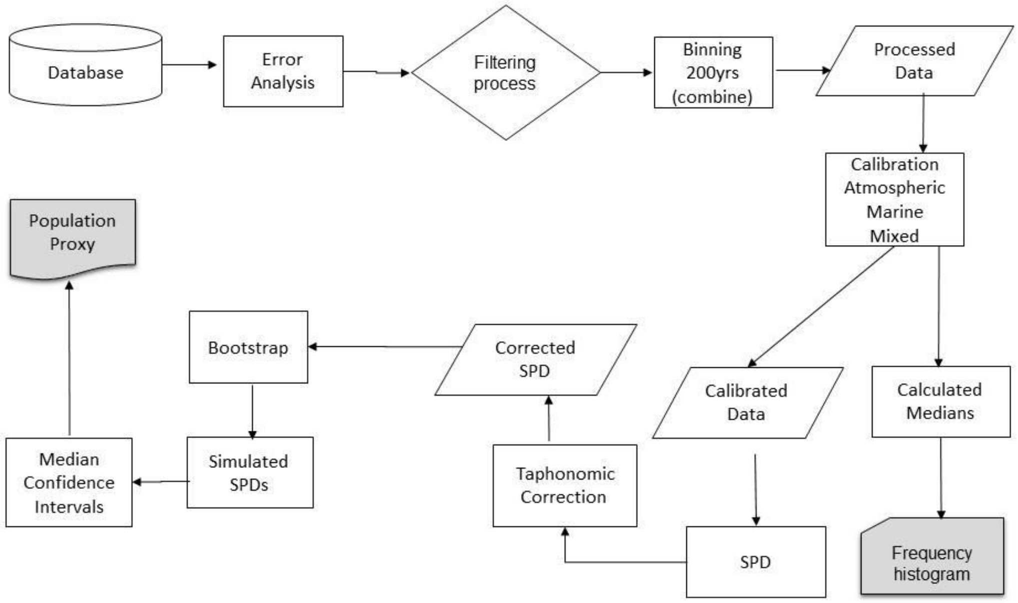

Supplementary Figure 5. Flowchart of the modelling approach followed in this study to obtain the SPD population proxy.

Recent works have brought to attention the effects of using normalized probability densities from individual dates, which consists in the rescaling of the area of the calibrated density distribution, before aggregating them into SPDs (26-28). Basically, the resulted SPD curve shows an exacerbation of spikes corresponding to the steepest parts of the calibration curve.

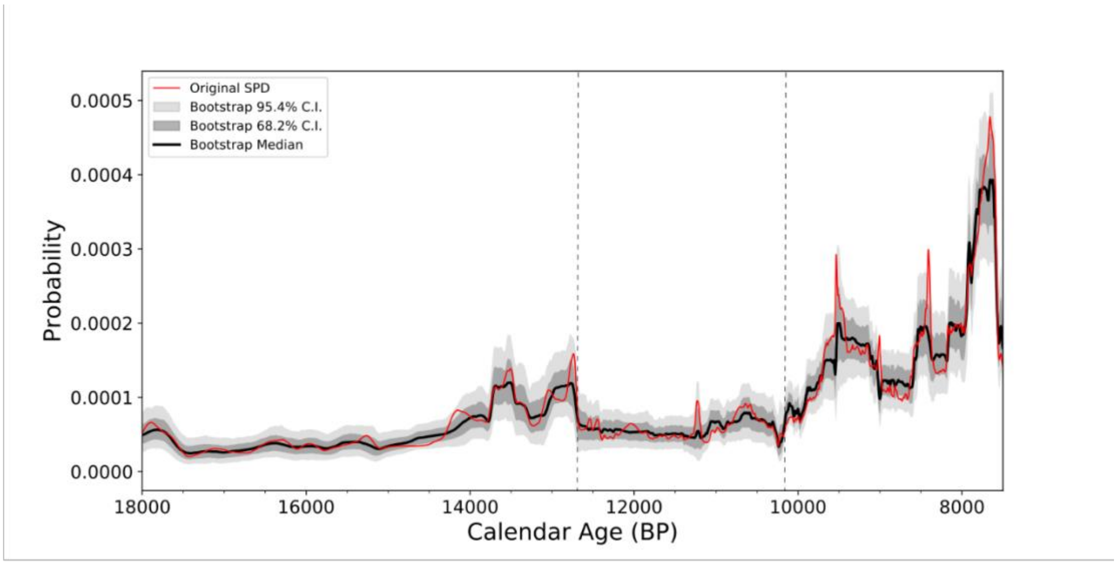

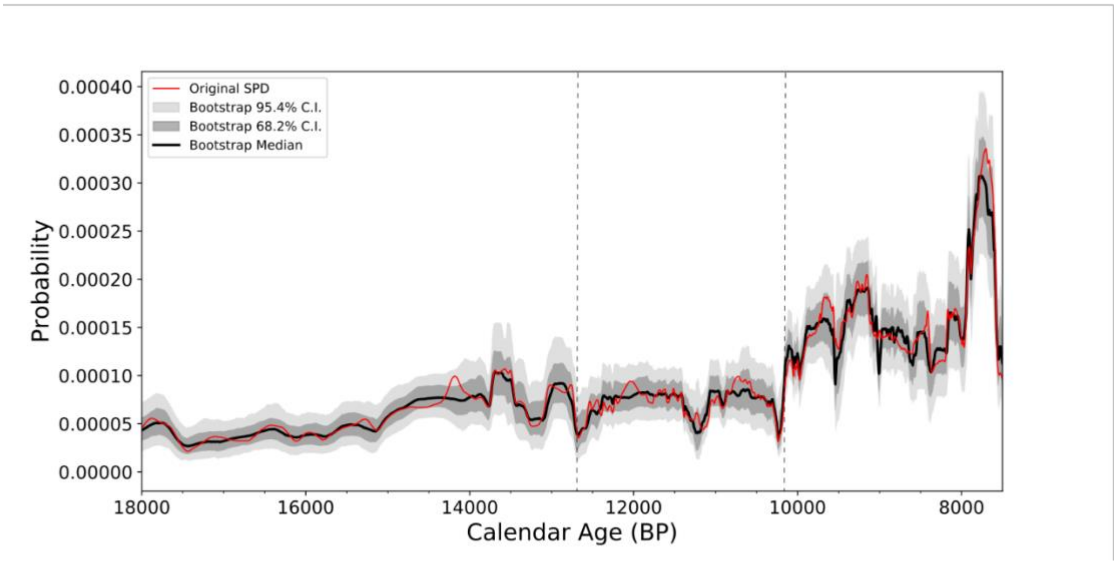

Supplementary Figure 6. Date normalization and its consequences on the analyzed time series. The red lines depict the original SPDs whereas the black bold lines depict the median of the bootstrap results. The vertical gray dashed lines represent the breakpoints between demographic phases. Top: SPD based on normalized dates after applying bootstrap and taphonomic bias correction. Bottom: SPD based on unnormalized dates. The observed long-term trends remain very similar despite differences in the density values of the transition between phase 1 and 2 (more attenuated when using unnormalized densities), in phase 2 (with slightly higher probability densities when using unnormalized) and the timing of the transition between phases 2 and 3 (more abrupt in the case of unnormalized than in normalized probability densities).

6.1 Taphonomic correction and non-linear scaling adjustment

The obtained SPD curve was then subjected to taphonomic bias correction using the Supplementary equation 1 proposed by Surovell *et al.* (25), which was here only applied to open-air sites.

$$n(t) = 5.726442 \cdot 10^{6} (t + 2176.4)^{-1,3925309} \quad (1)$$

where n(t) is the relative number of radiocarbon dates expected to survive from time t. The functions to produce Summed Probability Distributions (SPD) and apply the taphonomic bias correction were developed in Python and integrated with the IOSACal package. Both scripts are available on GitHub repository *PALEODEM/Palaeo-demographic-models* (as *SPD_curve.py* and *SPD_curve_taphonomic_correction.py*, respectively).

Freeman and colleagues have proposed a different scaling factor that aims to correct SPDs for the sub-linear relationship between energy consumption and population size they have observed for modern case studies (29). In Supplementary Figure 7, we compare the taphonomically corrected SPD for all the dates under analysis with an uncorrected curve and one re-scaled according to Freeman *et al.* (30). In the context of the current study, neither adjustment is particularly significant.

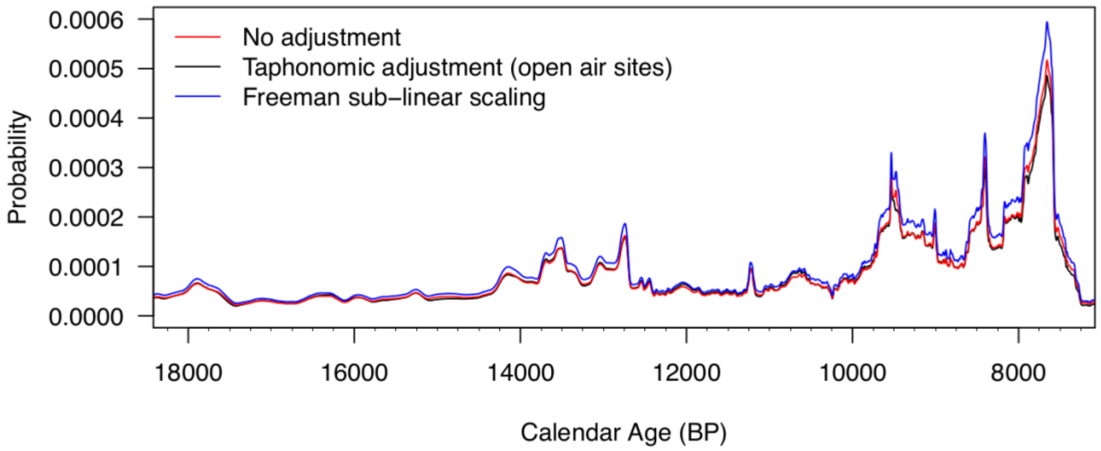

Supplementary Figure 7. Comparison between adjusted taphonomic or sublinear scaling and non-adjusted SPDs.

As Supplementary Figure 7 makes clear, taphonomic correction does not strongly influence the results of the current study. Between approximately 12,000 and 10,000 cal BP the taphonomically corrected SPD contains higher values than the uncorrected one, whereas at 8000 cal BP the uncorrected curve is higher. However, the differences are slight, and far below the level of statistical significance when considered alongside the bootstrapped confidence intervals. This is due to the archaeological context of the data in this study: most open-air sites in Iberia date from the Early Holocene and later. Before

10,000 cal BP, the vast majority of the dates are from caves or rockshelters, and although the Surovell correction would increase the summed probabilities for earlier periods (if all the dates were from open air sites), it is not applicable to the cave contexts considered for the current work.

The Freeman correction (Supplementary equation 2) has been applied to the uncorrected SPD (Supplementary Figure 7).

$$f'(t) = 1.15\, f(t) \qquad (2)$$

where f(t) is the SPD time series and f'(t) its scaled equivalent. Although this has as a more pronounced effect than the taphonomic correction, it is nonetheless a small adjustment, raising the level of the 8000 cal BP peak in the SPD but having little other effect. As discussed by Freeman and colleagues (29, 30), this correction works in the opposite sense to the taphonomic adjustment, having greater influence upon the later part of the study, for which the summed probability values are higher. As also pointed out by Freeman and colleagues, neither adjustment has much of an effect on small probability values. The Freeman adjustment is a preliminary model of true relationship between human population level and the production of archaeological materials, but its universal application is a matter for debate. It may predict a sub-linear relationship between the radiocarbon record and other archaeological proxy measures of population, but to the best of our knowledge, this is yet to be demonstrated empirically using archaeological data. Therefore, we have not applied it to the SPDs in the current study. Reads curious about now this adjustment affects the modelled growth rates fitted to the SPDs can revise our values by factor of 1.15.

6.2. Bootstrapping confidence intervals

Grounded on the criticism expressed about the validity of the SPD approach as a suitable population proxy, some authors have argued that other factors than population size may create peaks and troughs in summed probability distributions (31). In order to evaluate if the SPD curve in our case study, can be attributed to random sampling fluctuations introduced by the radiocarbon calibration curve or laboratory errors, instead of genuine changes in population densities, we have used bootstrapping simulation.

The application of this method allows to achieve two very important goals: Obtaining a population curve much less noisy than the original SPD and determining a confidence interval around our proxy. With the former, we improve the way to reduce the noise, usually consisting on applying a moving average whose consequence is dragging the spurious fluctuations along time, and with the latter, we introduce the ability to test whether fluctuations within the same curve, i.e. for different periods, are significant.

The application of bootstrap techniques at the radiocarbon dates time-series analyses, trace its roots back to the seminal paper of Rick (32) “Dates as Data”, and have

become very popular between a broad range of other scientific fields such as economics (33), or ecological fields (34). Focusing on archaeological and anthropological research, bootstrap resampling method has been recently used to show uncertainty in population density estimation (35), to test the statistical significance of the observed patterns in radiocarbon datasets analyzed (19), to examine sampling errors in datasets (36).

Bootstrapping is a standard statistical technique primarily utilized to provide a means of estimating standard errors of statistical estimates (37). The method consists essentially in assuming the original, and taphonomically corrected, SPD as a null model and obtain a back-calibrated set of dates analogous to the original one. We also assign the ^{14}C laboratory errors drawn from the original set so that the uncertainty associated to laboratory errors is actually the same. Then, we calibrate again the simulated set of dates to obtain a simulated SPD. The process can be repeated many times (in our study we ran up to 1000 iterations) in order to obtain a collection of simulated SPDs from which the median and 68% and 95% confidence intervals can be extracted. We consider this bootstrap median as our population proxy.

6.3 Required number of simulation runs

To test whether the number of bootstrap iterations was adequate, we repeated the process using different number of simulation runs. Visual inspection of the simulated SPD reveals a considerable amount of noise when only 10 sampling iterations are performed, but not much significant difference between the results for 100 and 1000 iterations (Supplementary Figure 8). This can be demonstrated more formally examining how the bootstrapping process converges upon a stable average (38). We have done this by subsetting the bootstrapping data and calculating the total difference between the median summed probability simulated each year over N sampling runs and the equivalent result after 1000 iterations. As shown in Supplementary Figure 9, stable simulations are reached quite quickly, after only approximately 50 sampling runs, and convergence is achieved after about 200 runs. Therefore, the 1000 sampling runs used throughout this paper can be seen as a conservative method that will not introduce noise or make a false representation of the data.

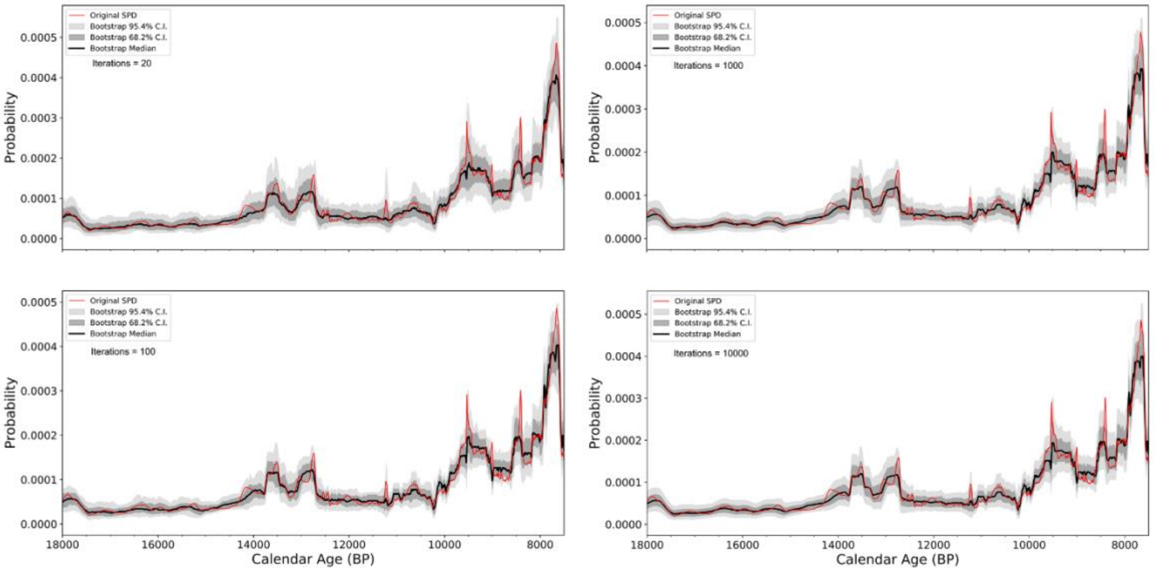

Supplementary Figure 8. Effect of the number of bootstrap simulations in the confidence interval of the SPD population proxy. Tests were performed with different number of iterations (N=10, N=100, N=1000, N=10000).

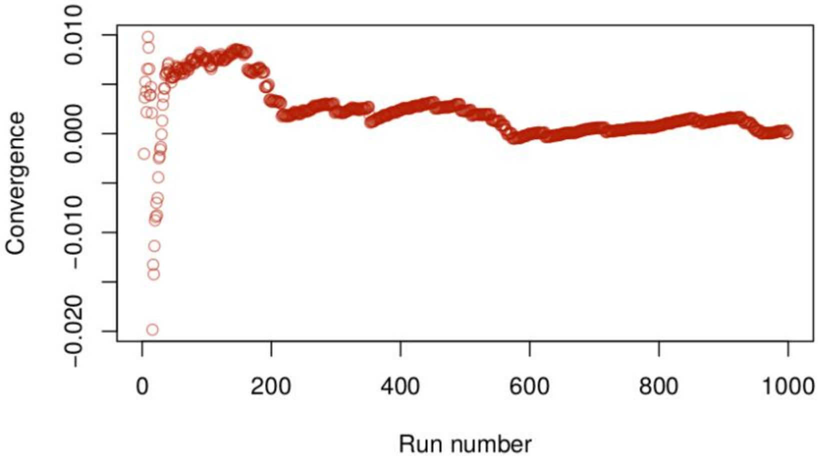

Supplementary Figure 9. Convergence analysis showing the average difference between 1000 simulated SPDs and their subsets.

The 8000 cal BP cut-off was also chosen to avoid 'edge effects' relating to potential interactions with the beginning of the Neolithic in Iberia. This period introduced to Iberia new cultural process that were not intrinsic to the dynamics of the hunter-gatherer societies that existed beforehand, and therefore not relevant for the purposes of our study. Furthermore, the timing of the start of the Neolithic was not uniform across Iberia, as it occurred later in Atlantic and Cantabrian regions than elsewhere. By limiting our model fitting analyses to events before 8000 cal BP we avoid these complexities and ensure our study is focused on endemic processes. This has the added advantage of excluding Mesolithic data post-dating 8000 cal BP from our analyses. These data derive from sites

that are highly visible, especially in the Atlantic region, and have been intensely investigated by archaeologists and thus represent an example of positive research bias.

6.4. Effects of the calibration curve (atmospheric or marine) on the observed SPD

In Supplementary Figure 10 SPDs of dates obtained from atmospheric and marine samples are compared to the overall SPD used for the analyses presented elsewhere in this paper. Samples from mixed carbon reservoirs, i.e. samples of human bone where stable isotopes have indicated a high level of marine protein in the diet, have been calibrated using an appropriate mixed curve and added to the atmospheric SPD.

Due to the lesser number of samples, the confidence intervals of the SPD of marine dates have a large span, and consequently they evince very little significant structure except for a pronounced spike at the end of the series (ca. 8000 cal BP). As Supplementary Figure 10 (bottom) makes clear, this spike in activity is due to the significant number of marine samples dating to the Late Mesolithic period. This, in turn, is a function of the nature of Late Mesolithic sites and the high visibility and long-standing research interest in shell middens and burials of the period (e.g. 39, 40).

Turning to the individual regional units (Fig.3 and Supplementary Discussion), it is therefore unsurprising that the 8000 cal BP spike is not present in any of the inland contexts. In short, this is an example of research bias influencing the shape of the SPDs, and for this reason we have excluded all points in time after 8000 cal BP from the model fitting exercise. Before this date, we can be more confident that the distribution of radiocarbon samples in time is randomized better and therefore more likely to reflect fluctuations in population. Marine samples are nonetheless important to include in the analyses overall because they have a subtle but significant influence on the SPD at other points in time, such as between 9500 and 9000 cal BP. Also noteworthy are periods of low activity common to both curves. Low activity levels between 10,500 and 10,000 cal BP is apparent in both atmospheric and marine SPDs, and the slight increase in activity associated with the start of demographic phase III is a common to both curves.

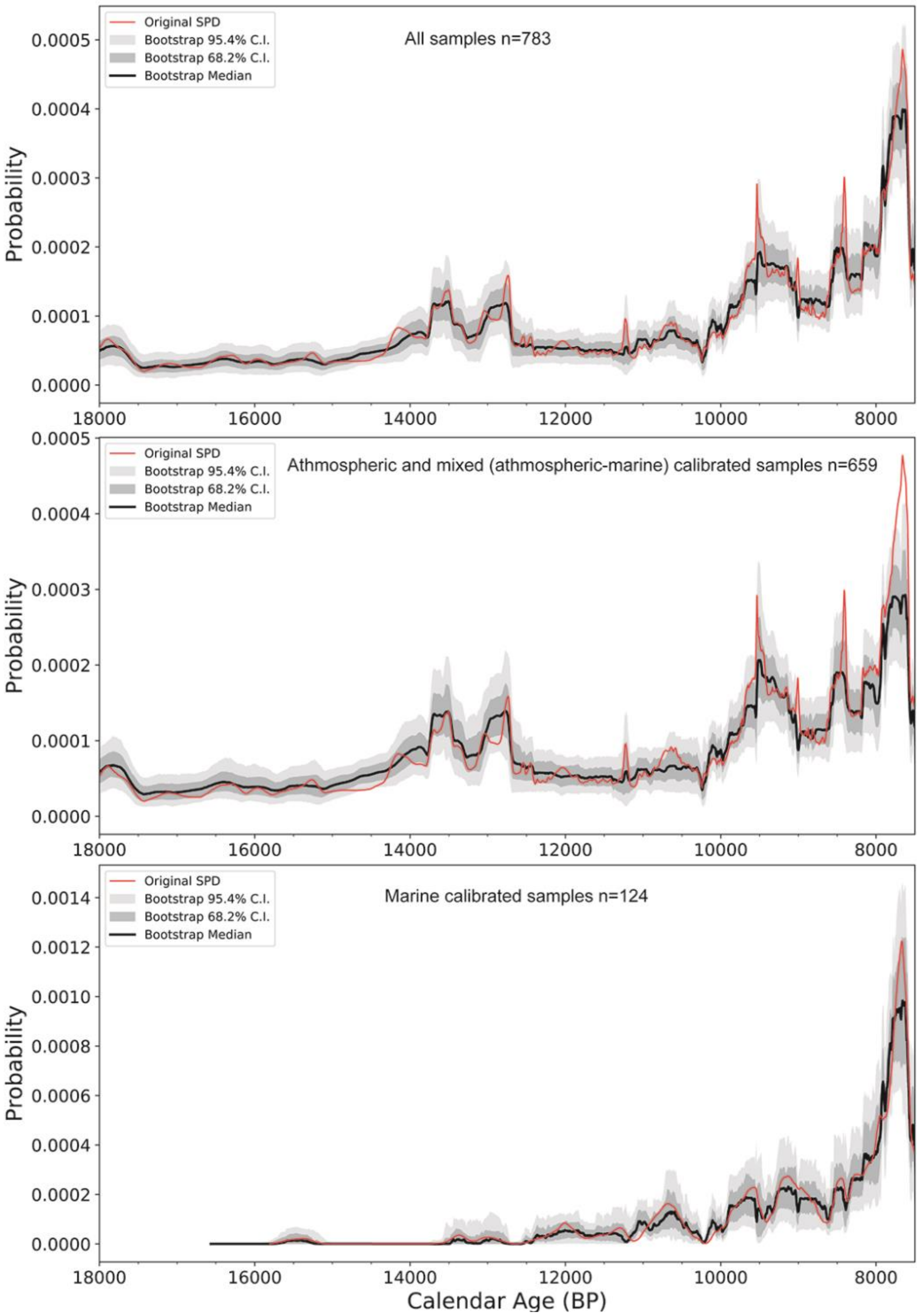

Supplementary Figure 10. A comparison of SPDs calculated using all 14C samples (top) atmospheric samples (middle) and marine samples (bottom).

6.5. Choice of calibration curves and back-calibration datasets for simulation

To ensure that the bootstrapping process used in this paper is robust against the choice of calibration curve used in the simulations, we have repeated the steps outlined above using different calibration curves for simulation. We have conducted the analysis using the atmospheric and marine Intcal13 curves, and by randomly allocating a specified proportion of the simulated dates to the marine ^{14}C axis when back-calibrating and re-calibrating. The results (Supplementary Figure 11) demonstrate that the choice of calibration curve for the simulations has no significant influence over the results. This, in-turn, implies that the stochasticity inherent to simulated SPDs is caused by the shape of the empirical SPDs and the number of data points or sampling locations in the analyses. In terms of calibration effects, it seems that the characteristics common to both the marine and atmospheric curves are more important to this process than the differences between them. The issue of simulating local reservoir effects is more complex. It is possible to draw from the source data, and assign these at random to the simulated dates, following the procedure used for assigning ^{14}C measurement error. However, because of the noncommutative nature the summing process, it is necessary to also apply a matching age offset to the samples drawn from the empirical SPD. This has two drawbacks: firstly, the age offsets are strictly not equivalent, since one is in calendar time and the other on the ^{14}C axis, but for practical purposes they are similar. Secondly, and more importantly, the random offset causes the process to integrate different parts of the calibration curve at either side of the sampling point, thereby imposing a smoothing or averaging effect upon the overall results at high frequencies. Conversely, as apparent in Supplementary Figure 11, there is also a tendency to sample more frequently from calibration curve spikes. The result is a set of bootstrap simulations that actually more closely resembles the original empirical SPD, but it does so by amplifying noise from the original calibration curve or curves from which the empirical SPD was calculated. Resolving the mathematical complexities of this situation is something we leave for future research. For the purposes of the present study, the choice of calibration curve for the simulations has demonstrably little influence.

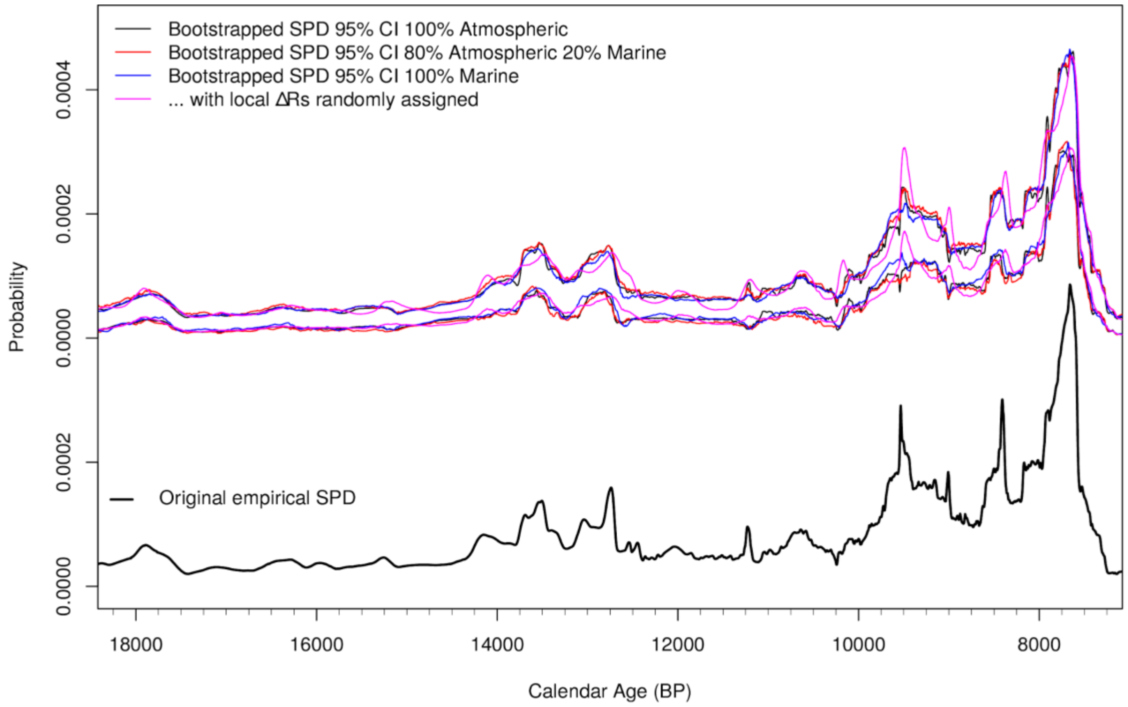

Supplementary Figure 11. A comparison of SPDs simulated using dates calibrated with the atmospheric (intcal13) curve, the marine13 curve, and a combination of dates where 80% were calibrated with the atmospheric curve and 20% with the marine curve (thus matching the relative proportion of dates in the original dataset). Also indicated is a curve composed of simulated dates calibrated using the marine curve, with added local reservoir effects drawn at random from the source data.

6.6. Testing a null model

To establish whether the observed relative population changes significantly deviate from a global trend, we have fitted an exponential function to the population proxy curve, thus creating our model for the null hypothesis. We have applied the bootstrapping methodology described above to the null model as well, in order to obtain 95.4% and 68.2% confidence intervals (CI) around the null hypothesis (script available in *PALEODEM/Palaeo-demographic-models* GitHub repository as *exponential_null_model_pvalue.py*). This technique allows to elucidate which local periods are above or below the 95.5% CI envelope (19,17).

Repeating this exercise using the marine curve for the bootstrapping instead of the atmospheric one, few differences occur in the various sets of results. This is true even where we have randomly applied local ΔR corrections during the simulations, sampling these from the source data. Compared with the results generated using the atmospheric intcal13 data (main text, Figure 2, bottom), there are no major differences concerning the periods where population is identified as significantly higher or lower than the null model (Supplementary Figure 12).

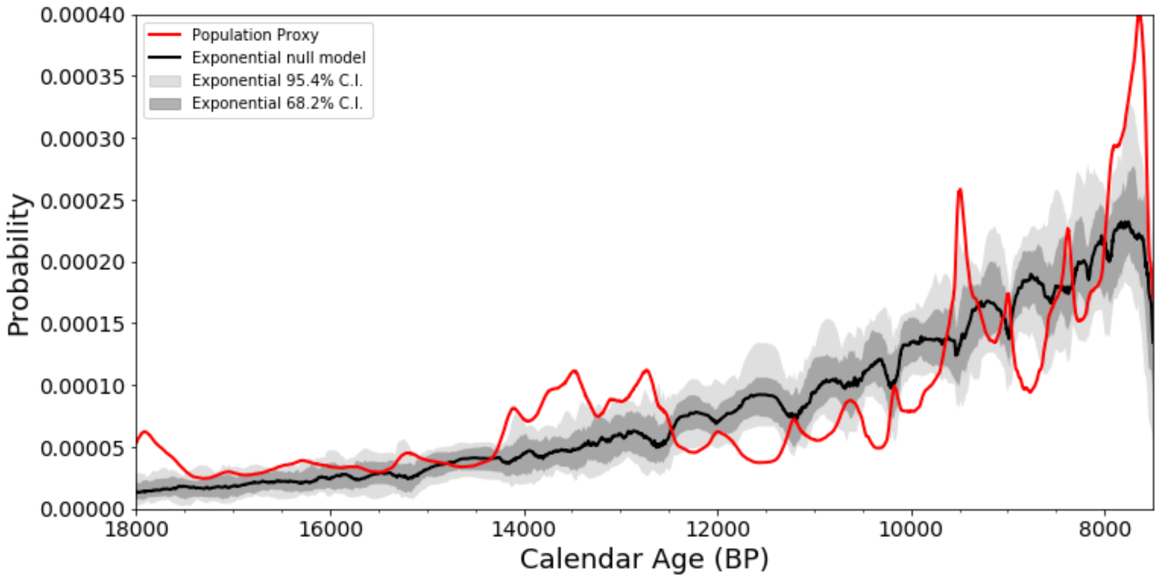

Supplementary Figure 12. A comparison of the simulated population proxy (median of 1000 simulation runs) and exponential null model (1000 simulation runs) calculated using the marine13 curve with local reservoir effects randomly sampled from the source data and applied to each simulated date. Global p<.000999

7. Model Selection and Demographic Growth Rates

7.1. Motivation and Choice of Demographic Model(s)

This section specifies the methodology followed in the model selection and calculation of the demographic growth rates discussed in the manuscript. A single exponential growth curve that fits the SPD for the entire time period of interest is the most widely used model in the literature (17, 19, 35, 36, 41). However, some authors, for some case-studies, have started to move beyond this paradigm. Bevan and collaborators (28) have used logistic growth for the Late Holocene of UK and Ireland; whereas Goldberg and collaborators (42) have suggested a model with two demographic phases for South America: a logistic growth phase, followed by exponential growth from the mid-Holocene onwards. In addition, Brown (43) has made the case for dynamic growth models derived from temporal frequency analyses.

In our case, as the null hypothesis significance testing found sufficient evidence to reject exponential growth as an explanatory framework for the population proxy, we have explored alternative and more complex hypotheses using an information-theoretic based model selection approach, which is explained in detail further below. Any model selection approach, however, must begin with a choice of models to test against the empirical data.

Rather than opting for a fully dynamic growth rate, as proposed by Brown (80), we opt for a more parsimonious solution, attempting to keep the number of changes in demographic regime to a minimum, whilst leaving more detailed work – which will inevitably involve analyses at the sub-regional level – for future work. We note that, to our

trained eye, there seem to be two natural breakpoints that coincide with well-established environmental and cultural transitions. The first one relates to the onset of the YD around 12.9 kya and the second with the onset of the Early Mesolithic around 10.2 kya. Our models, therefore, range from a single demographic phase to a total of three demographic phases. As is natural, the more phases are considered, the more variety in choice of functions to fit different phases. This choice was heuristic yet based on the shape of the population proxy, as well as on our own knowledge of the mathematical functions at hand. Of our six models, we considered two 2-phase models and three 3-phase models. The functions considered include both the exponential and logistic growth curves already considered in the literature as well as, for the 3-phase models, a logarithmic decay curve – whose shape is distinct from that of exponential decay – as well as an exponential decay towards a limiting value.

Our models, therefore, increase in complexity (Fig.4), from a single exponential or logistic fit to the entire temporal domain of interest (models A and B), to a dual exponential (C), dual logistic (D), a simple exponential for phases 1 and 3 with exponential decay towards a limiting value for phase 2 (E), and the same except for logistic growth fitted to phase 3 (F).

The choice of breakpoints between phases is very important since it will dictate whether or not a particular model will be a good fit to the data. For the present paper, we will consider only the two already mentioned breakpoints, namely that corresponding to the YD (12.9 kya) and the onset of the Mesolithic (10.2 kya). However, the timing of these transitions is not as sharp and well-dated as these often-cited dates seem to portray them. They also did not occur contemporaneously across the whole of the Iberian Peninsula. To account for this, we have allowed these breakpoints to vary by one-year increments within ranges given by the above-mentioned dates plus or minus 150 years. This will provide a measure of uncertainty around our inferences, especially the growth rates that should account for (at least some) sub-regional variability.

7.2. Model Selection Methodology

We expanded on the approach of Goldberg et al (42), where a 2-phase regression was fit to the SPD, the corresponding likelihood calculated and Schwarz Bayesian Criteria (SBC, also referred to as Bayesian Information Criteria or BIC) used to rank the different models. Unlike them, however, we decided to use Akaike's Information Criteria (AIC) which offers more robustness from both a theoretical and practical point of view (44, 45). In particular, AIC can also be derived from a Bayesian framework (with a savvy prior), but, unlike the SBC, it has a solid foundation in Information Theory. It is also demonstrably better than SBC at model selection in situations where there is a tapering effect with growing model complexity, as we expect the case to be with fitting a time series such as an SPD.

For each model, we picked the breakpoint values that yield the highest likelihood values and we then calculate the AIC index using the Supplementary equation 3.

$$AIC = -2\ log(L) + 2\ K\ , \qquad (3)$$

where $log(\mathcal{L})$ is the log-likelihood of the regression fit and $K$is the total number of parameters of the model, which is given by the number of regression parameters (two for the case of the exponential fit, three for the exponential with baseline and logarithmic fits and four for the logistic fit) plus one for the standard deviation, which is also estimated. The model with the lowest AIC value is the best-fitting one, and the AIC differences between the best model and the others can be used to calculate the weight of evidence in favor of each model, also known as the Akaike weights (45), and which are given by the Supplementary equation 4:

$$w_i = \frac{exp\left(-\frac{1}{2}\Delta_i\right)}{\Sigma_j \quad exp\left(-\frac{1}{2}\Delta_j\right)}\ , \qquad (4)$$

where Δ_i= AIC_i− $AIC_{min}$are the AIC differences for each model.

For completeness, we have also attempted to vary the beginning of our time range of interest in order to check whether this affected the best-fitting and model selection results. By doing this, one is effectively changing the dataset by adding extra data-points to the time series, whereas model selection criteria cannot be compared across different datasets. To overcome this issue, we ran our model selection routines for different values of the starting point separately, and compared results at the end. Interestingly, for each of these runs, the AIC (and indeed BIC) differences were the same irrespectively of the value of the starting point. This effectively means that, for model selection purposes, we can arbitrarily choose a starting point and, therefore, we have chosen the value of 16,600 cal BP which is the approximate date of the beginning of the Upper Magdalenian in the Cantabrian and the Mediterranean regions.

The modelling and selection routines were implemented in *R*, an open source programming language and software environment for statistical computing and graphics (46, https://www.R-project.org), using code which is freely available on GitHub (in repository *PALEODEM/Palaeo-demographic-models/Model_selection*).

7.3. Results

The best-fitting breakpoints for each model, corresponding to the ones already seen in Fig.4, along with corresponding selection criteria are shown in Supplementary Table 5. Of all the 90,601 possible combinations of breakpoint values, Model F was consistently the one with the lowest selection criteria, in addition to being the one with the absolute

lowest AIC and SBC values – which is why the weight of evidence is completely in favor of this model out of this set.

Model	Best-Fitting Breakpoints	AIC	SBC	Weight of Evidence
A	N/A	-156036.6	-156021.4	<0.001%
B	N/A	-158738.7	-158713.4	<0.001%
C	13,050	-160655.5	-160629.4	<0.001%
D	12,750	-165583.4	-165548.3	<0.001%
E	12,750 / 10,350	-166699.6	-166660.4	<0.001%
F	12,776 / 10,157	-168446.7	-168400.1	≈100%

Supplementary Table 5. Results of the model selection approach for all six models. The table reports the including best fitting breakpoint values, Akaike Information Criteria (AIC), Schwarz Bayesian Criteria (SBC) and weight of evidence as calculated using AIC differences.

7.4. Annual Growth Rates and Associated Uncertainty

Having identified the best-fitting model we set out to derive growth rates for the three demographic phases, and their associated uncertainties. Annual growth rates for phase 1 were simply calculated as (Supplementary equation 5):

$$r_{phase\ 1} = 100\ \cdot (exp(b) - 1)\ , \qquad (5)$$

where b is the exponential factor resulting from the SPD regression fitting. This equation cannot be applied for phases 2 and 3, because the growth rate of the fitted curve is not constant but changes with time. Instead, we calculated the dynamic growth rate from the fitted model numerically, using a general equation for growth rate (Supplementary equation 6):

$$r_{phases\ 2\ and\ 3}(t_i) = 100 \cdot \left(\frac{y(t_i)}{y(t_{i-1})} - 1 \right)\ , \qquad (6)$$

where $y(t_i)$ is the fitted curve value at time t_i. This equation is applicable to any fitted model.

Standard regression algorithms output uncertainty values that can be propagated into an uncertainty on the growth rate values. However, these underestimate the true uncertainty since they disregard other sources of uncertainty. One such source of

uncertainty, as mentioned above, relates to the choice of breakpoints. To estimate the uncertainty due to this we looked at all the runs of Model F for different breakpoint values, and obtained annual growth rates for each run. We then calculate their running mean and 95% confidence interval (CI), which are shown in Fig.5, where other relevant values reported in the literature are also plotted (36, 41). Mean, minimum and maximum growth rate values for the regions outside of the breakpoint ranges (inside of which they vary) are reported in Supplementary Table 6.

Annual Growth Rate	2.5%	Mean	97.5%
Phase 1	0.0402	0.0411	0.0420
Phase 2	-0.7908	$a\ exp(-b\ t) + c$<br>a = 4.54×10^{-24};<br>b = -0.00339;<br>c = 0.0000555	≈ 0
Phase 3	≈ 0	$A/(1+exp((x_0-t)/s)+c$<br>A = 0.000080<br>x_0 = 9799<br>s = 77.89<br>c = 0.000078	0.2278

Supplementary Table 6. Mean, minimum and maximum 95% confidence interval values for the annual growth rate of the three demographic phases, when taking breakpoint uncertainty into account.

Supplementary Discussion

8. Regional population patterns

The reconstruction of demographic trends at the Iberian scale is based on the aggregated analysis of a large ensemble of radiocarbon dates from several regional subsets with different research intensities. This section discusses the regional variability in the inferred relative population changes and to what extent the observed patterns might result from research and sampling biases. The investigation and cross-correlation of regional trends regarding regional paleoclimate and paleoenvironmental dynamics will be further analyzed and discussed in more detailed future works.

The Iberian Peninsula has been divided into 5 major regional subsets (Supplementary Figures 13 and 14): Cantabrian, Ebro Valley, Atlantic, Mediterranean and Interior. Such a division is based on regional analytical units well recognized by Iberian prehistorians (e.g. 47, 48) even though, in turn, each might equally be subject to some degree of sub-regional variation. In terms of biogeography, the whole Cantabrian subset falls within the Eurosiberian region whereas the rest of the regional subsets are mainly located in the Mediterranean domain, but under different sub-regional units depending on the continental influence and the altitude above the sea level (49). The main exception comes from Pyrenean areas, located in the north of Mediterranean and the Ebro Valley subsets, which corresponds to the Eurosiberian domain.

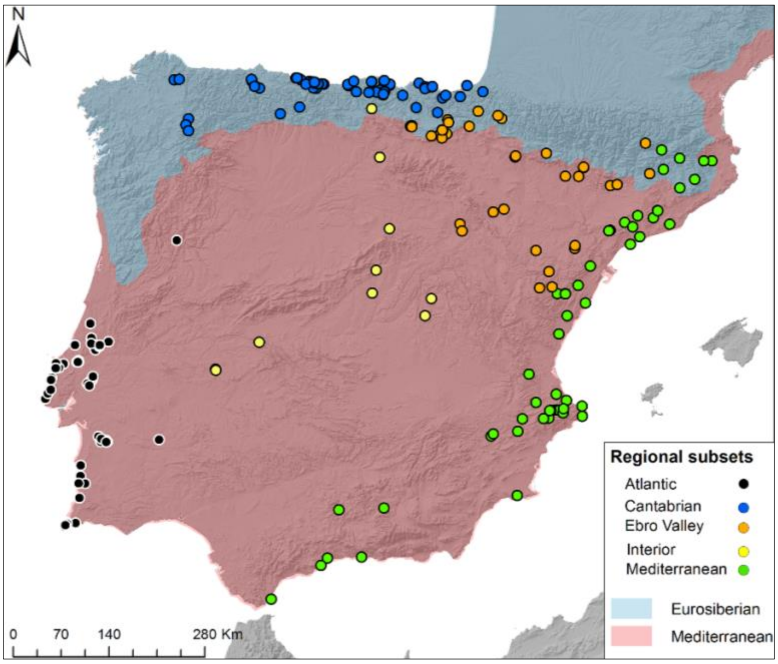

Supplementary Figure 13. Distribution of archaeological sites with radiocarbon dates split by regional subsets and bioclimatic regions (49). The background map has been generated in ArcGIS 10.3 using 1 arc-second SRTM raster data (available from https://earthdata.nasa.gov/) to represent terrain via a hillshade model.

In order to facilitate the correspondence between the long-term demographic patterns with the archaeological sequence, Supplementary Figure 14 displays the chrono-cultural scheme for each of the main regional subsets according to updated bibliographic references. This figure also represents the Greenland strato-type chronology (50) and the limits of the proposed demographic phases from the study of the growth rates (see Supplementary Methods Section 7).

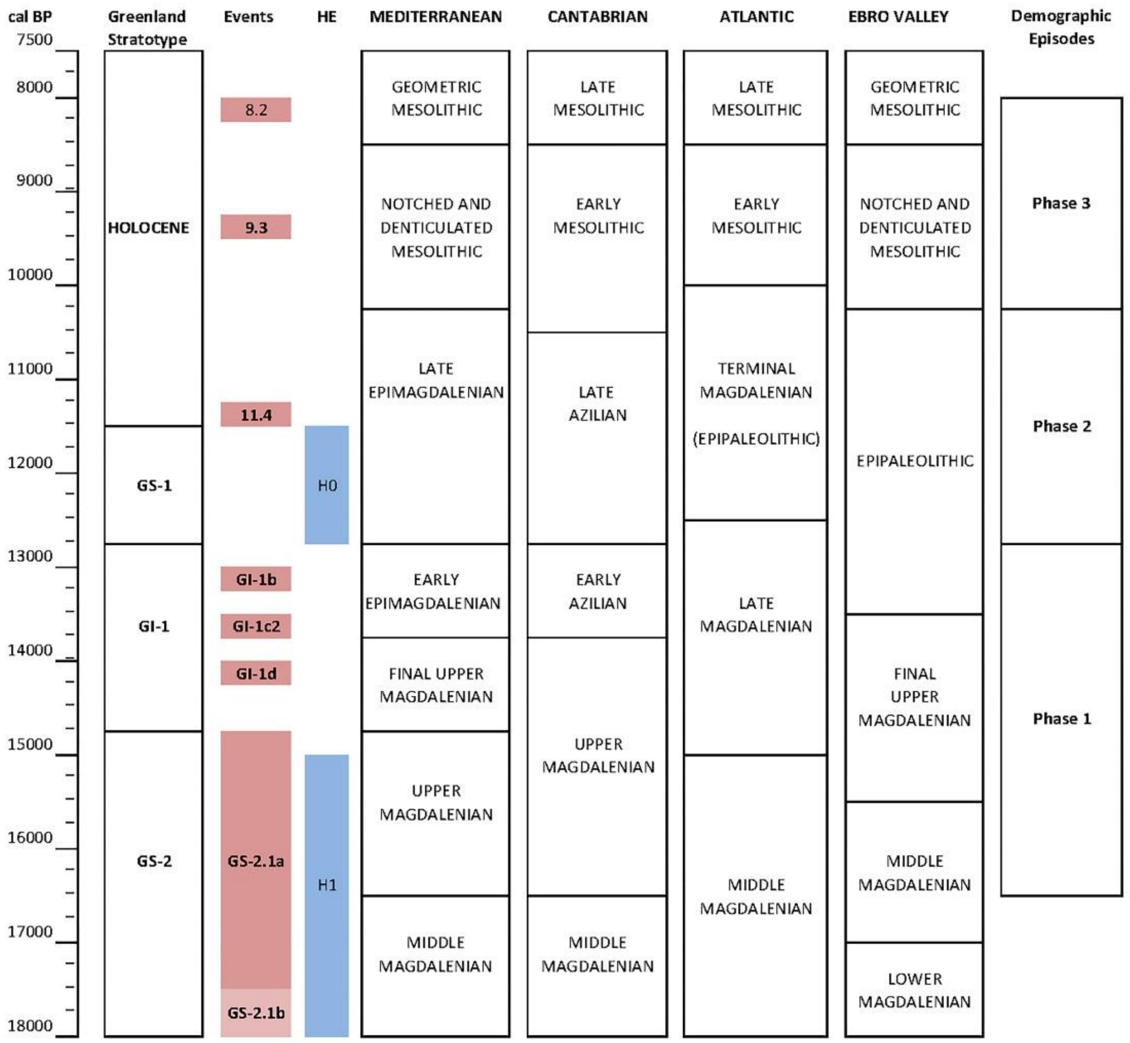

Supplementary Figure 14. Chrono-cultural scheme at the different regional subsets according to different authors: Mediterranean (51-53), Cantabrian (54), Atlantic (55) and the Ebro valley (56, 57). The right column displays its correlation with the demographic phases of the complex model of population dynamics.

For each regional subset, the radiocarbon record has been analyzed following the methodology outlined in the *Methods* section and described above (Supplementary Methods). Subsequently, the population proxy was rescaled and compared to the 95% confidence interval (CI) of a null model of exponential growth. To fit this exponential null model on each regional population proxy, we have generated a randomly subsampled file

from the original (Iberian) dataset, with the same number of radiocarbon dates than the original regional unit dataset. Such a comparison allows to identify, in each regional time series, both positive and negative departures from the exponential model of demographic growth.

8.1. Cantabrian subset

In the Cantabrian subset, the population proxy shows four episodes with regional population densities above the exponential null model. The first two occurred virtually in succession along the Middle Magdalenian (18-17.6 kya and 17.4-16.3kya) and the first half of the Upper Magdalenian (15.6-15.3 kya) (58), during Heinrich event 1. After this episode, the population levels slightly decreased falling within the 95% CI of the null exponential model. A subsequent abrupt increase in the population proxy is observed at the end of the Final Upper Magdalenian leading to a short episode, around 13.7-13.5kya, when the inferred population lies above the exponential envelope. This episode roughly corresponds to the transition from the Final Magdalenian to the Early Azilian, after which we identify a consistent downward trend in population densities, between the end of GI-1 and the Younger Dryas to fall below the exponential null model during two negative pulses along the Early Holocene at 11.7-11.3,9.3-86. kya. Finally, there is a recovery in the population proxy to reach a brief peak at 7.7-7.65 kya, during the Late Mesolithic period.

Certainly, there are some research and taphonomic biases that could have affected the observed patterns. Firstly, a considerable number of Upper Magdalenian radiocarbon dates come from archaeological cave deposits discovered and partially excavated in the early 20th century. In those sites, there is an under-representation of the Early Holocene archaeological record, which was partially exhumed to reach the richer Upper Palaeolithic deposits. Systematic fieldwork projects based on karstic complexes such as El Mirón and La Garma, firmly provide Early Holocene occupational evidence (59-61) even though without the densities of faunal and lithic remains found in previous Middle to Upper Magdalenian, characterised by dense palimpsest deposits (62).

In addition, new dating programs focused on Asturian (Early and Late Mesolithic) deposits tend to correct this bias in the radiocarbon record (63, 64). A second problem is the destruction of archaeological sites produced by the sea level rise, which certainly should have affected the coastal Late Upper Paleolithic and Early Holocene sites. For the Early Holocene section, especially for the Asturian period, we note a positive research bias on shell midden research which has produced a considerable number of radiocarbon dates on shell samples (n=25) and human remains requiring a marine and mixed marine and atmospheric calibration (12, 65).

8.2. Ebro Valley subset

The Ebro valley time series presents three positive and one negative deviations from the exponential null model, reflecting three phases of statistically significant higher population levels. The first one is observed during 14.2-13.8 kya (Final Upper Magdalenian), leading to a statistically significant positive deviation and, a second isolated pulse around 13.6 kya, during the transition from the Late Upper Magdalenian to the Epipaleolithic. From this point, we observe a gradual decrease on the population proxy leading to a short episode 11.8-11.5kya below the 95.4% CI. A gradual monotonic increase in the population proxy is documented from 11.2 kya, leading to the second episode 8.7-8.5kya in which the population proxy increases above the 95.4% CI, roughly corresponding to the beginning of the Late Mesolithic. A sharp decrease in the SPD is documented from the 8.4 to the 8 kya, which could be correlated with the effects of the 8.2 kya cold climatic event on Late Mesolithic populations (66, 67). Such decrease, however, has not produced any statistically significant deviation from the exponential null model.

Even though the sample size from this regional subset seems representative enough for drawing major trends on relative population changes, there are several research and sampling biases, which could potentially affect the observed patterns. Firstly, most of the radiocarbon record analyzed in this regional unit comes from rock shelter-oriented fieldwork, focused on multi-layered archaeological deposits (68). In some subregional subsets, like the Central Ebro Valley, the archaeological record is mainly composed of rock shelters sites, with stratigraphic evidence dated to the second half of the Early Holocene, corresponding to the Early and Late Mesolithic periods (69, 70). In addition, in this area, different research programs have focused on the Mesolithic to Neolithic transition or on the archaeological context of the Levantine rock art, both resulting in an overrepresentation of Late Mesolithic radiocarbon dates. Certainly, more recent research programs focused on rock shelter sites have provided occupational evidence from the Early Magdalenian to the end of the Late Mesolithic (56, 57) including Younger Dryas-Early Holocene occupations.

8.3. Mediterranean subset

The Mediterranean subset shows two main episodes at 13.7-13.6kya and 13.1-12.7 kya with population levels above the 95.4% CI of the exponential model of demographic growth. These regimes of positive growth cover most of the Final Upper Magdalenian and the Early Epimagdalenian periods.

In contrast, we find an abrupt decrease in the relative population proxy chronologically correlated with the onset of the YD in the Mediterranean region (71, 72). From here, the inferred population levels show a steady fluctuating pattern within the broad 95.5% CI envelope of the null exponential model, until the end of the time series.

While the general pattern described by the population proxy is very similar to other neighbor units such as the Ebro Valley, it is worthy to note some research biases. First, the Final Upper Magdalenian and the succeeding Epimagdalenian have been intensively studied, focusing most of the fieldwork effort, which has produced datable material (52, 53, 73, 74). This, in part, might explain the overrepresentation of dates from the final part of GI-1 causing an abrupt increase in population proxy. In addition, the regional subset is affected by certain degree of sub-regional variation, which is especially meaningful for the Late Mesolithic, a period whose archaeological evidence remains elusive in Cataluña (75,76).

8.4. Atlantic subset

In the Atlantic subset, most of the current radiocarbon record comes from the central and southern regions of Portugal, whereas the northern area (including the well-known Côa Valley) has produced very few radiocarbon dates, most of them from Early Holocene occupations (77).

The Atlantic subset fits the expectations of the exponential model, showing two major positive deviations at 9.4-9.3kya, during the Early Mesolithic and, particularly, at 7.9-7.5kya during the Late Mesolithic. For most of the time series, however, the reconstructed population is within the 95.4% CI limits of the exponential model, with three episodes of statistically significant negative departures from the null exponential model, one of longer duration at 15.2-14.3kya, and two more exiguous pulses around 12.7 kya and 10.4 kya.

Previous studies have suggested the impact of the Heinrich 1 stadial (18-15.6 kya) and the YD (12.8-11.6 kya) on population densities (55), which is consistent with our results.

The Preboreal-Boreal transition shows a consistent upward trend leading to a period, between 9.5-9 kya, of statistically-significant high population densities. This trend falls within the so-called Early Mesolithic in which human groups broaden their diets incorporating well adapted forest game and small prey (78). As mentioned above, the second episode of significant population increase is dated during the Late Mesolithic, a period of major transformations on settlement and subsistence patterns. Different studies suggest a major relationship between the regional effects of the 8.2 kya cal BP event with a change of the upwelling conditions and the increase of shellfish productivity favoring the settlement concentration in estuaries such as those of the Muge and Sado rivers (39, 79). The appearance of shell middens associated with Mesolithic cemeteries suggests a shift to more stable residential sites in ecologically rich estuaries (39). Another issue is determining to what extent the higher residential stability inferred from these shell middens corresponds to permanent or recurrent occupations as recently addressed in pioneering high-resolution micro-stratigraphic studies (80).

It is noteworthy that the increase of the population proxy observed during the Early Holocene, specially for the Late Mesolithic, could be partially explained by a positive research bias on the shell middens research in the Muge and Sado estuaries or the Western Algarve (40, 81, 82). In fact, the Atlantic subset concentrates the highest number of

radiocarbon dates requiring marine calibration, both on shell samples (n=119) and or human remains with a variable percentage of marine diet (n=25).

8.5. Interior subset

The interior subset shows a highly discontinuous trend caused by the lower intensity of the archaeological research compared to the other regional subsets included in this study. Even though the low number of dates and sites currently available for this area prevents us from obtaining reliable estimates of relative changes on population densities, it is worthy to mention the major continuities and discontinuities inferred by the radiocarbon record considered in recent works.

While there is stratigraphically and chronometric consistent information for the Middle and Upper Magdalenian (83, 84) we observe two major periods without reliable radiocarbon evidence, one dated 12.4-10.3 kya during most of the Younger Dryas and the Preboreal, and another from the 8.5 kya onwards.

9. Correlation with proxy records of palaeoenvironmental change

To quantify the degree of correlation between the Iberian population model and proxy indicators of environmental and climate change, we use Spearman's rank correlation test. We feel this is useful approach for describing the overall responses of human population to changing environmental conditions. The climatic and biogeographic diversity of the Iberian Peninsula hampers attempts to study the causal relationships between population levels and environmental change, for example using cross-correlation techniques. This work should instead be undertaken at a sub-regional level, where such relations hold meaning. Such further work is planned; what we report here is a descriptive account that such relations exist in Iberia and are worthy of future investigation.

Taking advantage of methods whereby we have bootstrap-simulated SPDs, we perform the correlation test 1000 times for each proxy, each time using a different simulated SPD. In this way, we can express what variability in the resulting correlation coefficients is caused by errors inherent to the process of radiocarbon measurement and calibration.

The palaeoenvironmental proxies considered here are temperature globally, via GISP2 18O records (50) and regionally via a sea surface temperature proxy from the deep-sea core MD95-2043 in the Alborán Sea (71) and the pollen-based indexes of precipitation and temperate Mediterranean forest (72). These are plotted alongside the SPD bootstraps in Supplementary Figure 18.

The results indicate that overall, by considering the period 18,000 to 8000 cal BP, there are strong correlations between all the palaeoenvironmental proxies and the demographic proxy. During demographic phase 1, we find a strong correlation between

vegetation, temperature and precipitation records. During demographic phase 2, we observe no significant correlations. During demographic phase 3, the only significant correlation in the data is a weakly negative correlation between global temperature and demography. These results are summarized in Supplementary Table 7.

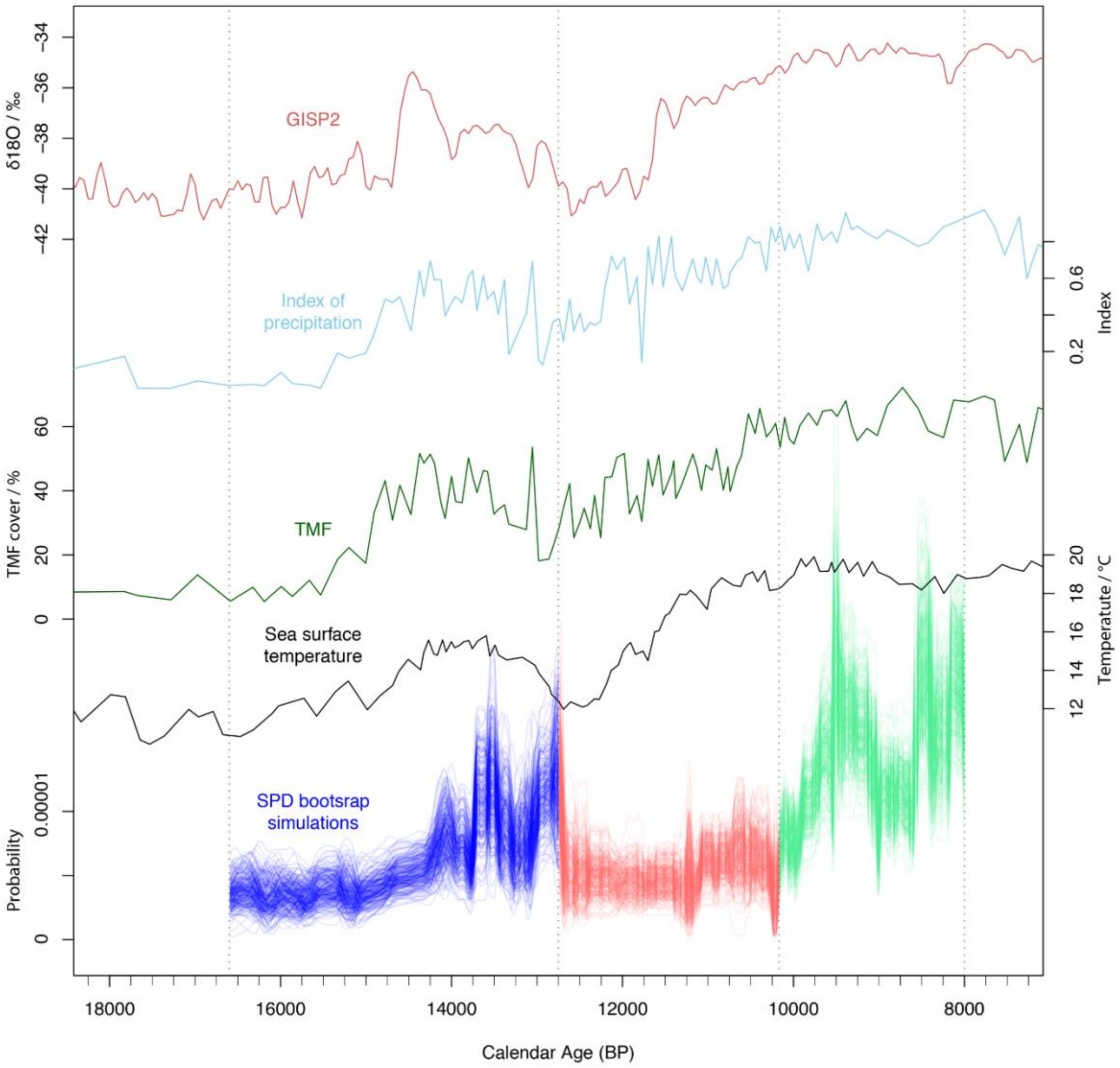

Supplementary Figure 15. Climate and demographic proxy data used for correlation analysis. Top (red line): Variation of $\delta^{18}O$ values from the GISP2 ice-core record (50). Middle (turquoise line): Precipitation index (I_p) based on pollen data from Alboran Sea marine core MD95-2043 (72). Middle (dark green line): Percentage of Temperate Mediterranean Forest (TMF) cover, based on pollen indices from Alboran Sea marine core MD95-2043 (72). Middle (black line): Sea Surface Temperature (SST) based on Alkenone from core MD95-2043 (71). Bottom: SPD bootstrap simulations employed at the Spearman's rank correlation coefficient analysis. Colours denote the demographic phases considered at this study: Demographic Phase I (blue), Demographic Phase II (light red), and Demographic Phase III (light green).

Proxy	Overall (18,000 to 8000 cal BP)	Demographic Phase I	Demographic Phase II	Demographic Phase III
GISP2 $\delta^{18}O$	0.67±0.14 $p<10^{-13}$	0.53±0.14 $p<10^{-13}$	0.15±.028 p≈0.005	-0.1±0.14 p≈0.005
Sea surface temperature	0.58±0.12 $p\approx 7.3\times 10^{-12}$	0.55±0.12 $p<10^{-13}$	0.12±0.28 p≈0.15	0.05±0.17 p≈0.59
Index of precipitation	0.46±.1 $p\approx 3.7\times 10^{-7}$	0.38±0.1 p≈.01	-0.04±0.21 p≈0.35	0.21±0.12 p≈0.34
Temperate Mediterranean forest (TMF)	0.47±0.1 $p\approx 1.7\times 10^{-7}$	0.35±0.11 p≈0.02	0±0.22 p≈0.35	0.32±0.15 p≈0.13

Supplementary Table 7. Correlation coefficient (Spearman's *ρ*) for proxy data and 1000 bootstrapped SPDs (mean±s.d., normally distributed) * – from 13,000 to 8000 cal BP. p values (median value for the 1000 iterations) indicate the probability of the correlation being due to random chance.

Like any correlation, care must be taken not to over-interpret these results. They serve a descriptive function; overall, increased temperatures, precipitation and vegetation associated with the Pleistocene-Holocene transition should be expected to positively correlate with demographic increases. Turning to the separate demographic phases, a similar correlation should be expected during Demographic Phase I, as (notwithstanding the onset of the Younger Dryas at the end of the phase), on average, the climatic conditions were improving and the human population was growing steadily. Conversely, the fluctuating population during Demographic Phase III is clearly at odds with the stable environment of the Early Holocene, and as such, correlation is neither expected nor observed.

This exercise usefully highlights the special properties of Demographic Phase II. There are no correlations during this phase because whilst the environment was undergoing rapid change, the human population remained stable and low (as modelled in our study as an exponential fall at ca. 12,750 BC towards a limiting factor). At this scale of analysis, constant changes to the environment could in theory have imposed a carrying capacity significantly less than the circumstances prevalent at the end of Demographic

Phase I. As this exercise demonstrates, the archaeological 14C data are consistent with this hypothesis.

As an aside, it is noteworthy that one of the fluctuations during demographic phase III, a temporary downturn in activity during the late 9th millennium, appears to coincide with the '8.2 kiloyear event'. However, it should be noted that the downward movement of the radiocarbon record actually occurs before the 8.2k event, between 8400 and 8200 cal BP, and a causal relationship with the 8.2k event cannot be inferred.

Differences in environmental conditions between Demographic Phases II and III are highlighted when we compare the variance of the palaeoenviromental proxy data during each phase. To express this numerically, we have detrended the proxy data by fitting a composite series of linear models (one per demographic phase) to the time series, subtracting these functions from the proxy data, and calculating the variance of the result. We used the 'detrend' function in the R package 'pracma' to do this. The results are shown in Supplementary Table 8.

Proxy	Demographic Phase I	Demographic Phase II	Demographic Phase III
GISP2 $\delta^{18}O$	1.3‰	0.43‰	0.11‰
Sea surface temperature	1.3 °C	0.59 °C	0.24 °C
Index of precipitation	0.038	0.017	0.0043
Temperate Mediterranean forest (TMF)	180%	46%	23%

Supplementary Table 8. Detrended variance (σ2) of the palaeoenvironmental proxies during each demographic phase.

In all cases, the palaeoenvironmental proxies have much less variance during Demographic Phase III compared to Demographic Phase II, modelling how instability in the environment can be a constraining factor for human populations.

Of course, during Demographic Phase I, the environment was even more unstable, but because in Iberia the demographic response was better correlated with generally increasing temperatures and precipitation (Supplementary Table 7), there was less impact on population. From 14,000 cal BP, as Younger Dryas environmental change took hold, a complex interplay between environment and population can be observed, again reflecting the general pattern that environmental instability is a limiting factor for human population growth.

Supplementary References

1. Waterbolk, H. T. Working with radiocarbon dates. *Proc. Prehist. Soc.* **37**, 15-33 (1971).
2. Pettitt, P., Davies, W., Gamble, C. S. & Richards, M. B. Palaeolithic radiocarbon chronology: quantifying our confidence beyond two half-lives. *J. Archaeol. Sci.* **30**, 1685-1693 (2003).
3. Wood, R. From convention to revolution: the past, present and future of radiocarbon dating. *J. Archaeol. Sci.* **56**, 61-72 (2015).
4. Zilhão, J. Radiocarbon evidence for maritime pioneer colonization at the origins of farming in west Mediterranean Europe. *Proc. Natl Acad. Sci. USA* **98**, 14180-14185 (2001).
5. Lanting, J. N., Aerts-Bijma, A. T. & Van Der Plicht, J. Dating of cremated bones. *Radiocarbon* **43**, 249-254 (2001).
6. Van Strydonck, M., Boudin, M. & De Mulder, G. ^{14}C dating of cremated bones: The issue of sample contamination. *Radiocarbon* **51**, 553-568 (2001).
7. Zazzo, A., Saliège, J-F., Person, A. & Boucher, H. Radiocarbon dating of calcined bones: Where does the carbon come from? *Radiocarbon* **51**, 601-611 (2009).
8. Brock, F., Bronk Ramsey, C. & Higham, T. Quality assurance of ultrafiltered bone dating. *Radiocarbon* **49**, 187-192 (2007).
9. Higham, T. F. G., Jacobi, R. M. & Bronk Ramsey, C. AMS Radiocarbon dating of ancient bone using ultrafiltration. *Radiocarbon* **48**, 179-195 (2006).
10. Monge-Soares, A.M. & Alveirinho Dias, J.M. Coastal upwelling and radiocarbon evidence for temporal fluctuations in ocean reservoir effect off Portugal during the Holocene. *Radiocarbon* **48**, 45-60 (2006).
11. Cook, G. T. et al. Best Practice Methodology for 14C Calibration of Marine and Mixed Terrestrial/Marine Samples. *Quaternary Geochronology* **27**, doi:10.1016/j.quageo.2015.02.024 (2015).
12. Monge-Soares, A.M. *et al.* Marine radiocarbon reservoir effect in Late Pleistocene and Early Holocene coastal waters off Northern Iberia. *Radiocarbon* **58**, 869-883 (2016).
13. Fano, M.A., Cubas, M. & Wood, R. The first farmers in Cantabrian Spain: Contributions of numerical chronology to understand an historical process, *Quat. Int.* **364**, 153-161 (2015).
14. Goodfriend, G.A. & Hood, D.G. Carbon isotope analysis of land snail shells: implications for carbon sources and radiocarbon dating. *Radiocarbon* **25**, 810–830 (1983).
15. Yanes, Y., Gómez, M., Esquembre, M.A. & Fernández-López de Pablo, J. Younger Dryas – early Holocene transition in the south-eastern Iberian Peninsula: insights from land snail shell middens. *J. Quat. Sci.* **28**, 777-788 (2013).

16. Collard, M., Edinborough, K., Shennan, S. & Thomas, M.G. Radiocarbon evidence indicates that migrants introduced farming to Britain. *J. Archaeol. Sci.* **37**, 866-870 (2010).
17. Crema, E., Habu, J., Kobayashi, K. & Madella, M. Summed Probability Distribution of ^{14}C dates suggests regional divergences in the population dynamics of the Jomon Period in Eastern Japan. *PLoS ONE* **11**, e0154809. doi:10.1371/journal.pone.0154809 (2016).
18. Peros, M.C., Munoz, S.E., Gajewski, K. & Viau, A.E. Prehistoric demography of North America inferred from radiocarbon data. *J. Archaeol. Sci.* **37**, 656-664 (2010).
19. Timpson, A. et al. Reconstructing regional population fluctuations in the European Neolithic using radiocarbon dates: a new case-study using an improved method. *J. Archaeol. Sci.* **52**, 549-557 (2014).
20. Ward, G. K. & Wilson, S. R. Procedures for Comparing and Combining Radiocarbon Age-Determinations - Critique. *Archaeometry* **20**, 19-31 (1978).
21. Reimer, P.J. et al. IntCal13 and Marine13 Radiocarbon Age Calibration Curves 0-50,000 Years cal BP. *Radiocarbon* **55**, 1869-1887 (2013).
22. Reimer, P. J. & Reimer, R. W. A marine reservoir correction database and on-line interface. *Radiocarbon* **43**, 461-463 (2001) (http://www.calib.org).
23. Ascough, P.L., Cook, G.T. & Dugmore, A.J. Methodological approaches to determining the marine radiocarbon reservoir effect. *Progress in Physical Geography* **29**,532–547 (2005).
24. Martindale, A. *et al*. Estimating marine reservoir effects in archaeological chronologies: Comparing ΔR calculations in Prince Rupert Harbour, British Columbia, Canada. *Am. Antiq.* **83**, 659-680 (2018).
25. Surovell, T. A., Finley, J., Smith, G. M., Brantingham, P & Kelly R. Correcting temporal frequency distributions for taphonomic bias. *J. Archaeol. Sci.* **36**, 1715-1724 (2009).
26. Michczynski, A. & Michczynska, D.J. The effect of PDF peaks' height increase during calibration of radiocarbon date sets. *Geochronometria* **25**, 1-4 (2006).
27. Weninger, B., Clare, L., Jöris, O., Jung, R. & Edinborough, K. Quantum theory of radiocarbon calibration. *World Archaeol.* **47**, 543-566 (2015).
28. Bevan, A. *et al*. Holocene fluctuations in human population demonstrate repeated links to food production and climate. *Proc. Natl Acad. Sci. USA* **114**, E10524-E10531 (2017).
29. Freeman, J. *et al*. Synchronization of energy consumption by human societies throughout the Holocene. *Proc. Natl Acad. Sci. USA* **115**, 9962-9967 (2018).
30. Freeman, J., Byers, D.A, Robinson, E. & Kelly, R.L. Culture process and the interpretation of radiocarbon data. *Radiocarbon* **60**, 453-467 (2018).
31. Contreras, D. A. & Meadows, J. Summed radiocarbon calibrations as a population proxy: a critical evaluation using a realistic simulation approach. *J. Archaeol. Sci.* **52**, 591-608 (2014).

32. Rick, J.W. Dates as Data: An examination of the Peruvian Preceramic Radiocarbon Record. *Am. Ant.* **52**, 55-73 (1987).
33. Khim-Sen Liew, V. *An Overview on Various Ways of Bootstrap Methods*. Munich Personal RePEc Archive (repository).Paper No.7163 (2008).
34. Nahorniak, M., Larsen, D.P., Volk, C. & Jordan, C.E. Using Inverse Probability Bootstrap Sampling to Eliminate Sample Induced Bias in Model Based Analysis of Unequal Probability Samples. PLoS ONE 10(6): e0131765. doi:10.1371/journal.pone.0131765 (2015).
35. Shennan, S. *et al.* Regional population collapse followed initial agriculture booms in mid-Holocene Europe. *Nat. Commun.* **4**:2486 doi: 10.1038/ncomms3486 (2013).
36. Zahid, H. J., Robinson, E. & Kelly, R. L. Agriculture, population growth, and statistical analysis of the radiocarbon record. *Proc. Natl Acad. Sci. USA* **113**, 931-935 (2016).
37. Efron, B. Bootstrap Methods: Another Look at the Jackknife. The Annals of Statistics **7**, 1-26 (1979).
38. Beran, R. Diagnosing Bootstrap Success *Annals of the Institute of Statistical Mathematics* **49**, 1–24 (1997).
39. Bicho, N., Umbelino, C., Detry, C. & Pereira, T. The emergence of Muge Mesolithic shell middens in Central Portugal and the 8200 cal yr BP Cold Event. *Journal of Island and Coastal Archaeology* **5**, 86-104 (2010).
40. Nukushina, D. The Mesolithic-Neolithic transition through the Iberian shell middens. A review of the current evidence and interpretations. In Coelho, I., Torres, J., Gil, L. and Ramos T. (eds.) *Entre Ciência e Cultura: Da Interdisciplinaridade à Transversalidade da Arqueologia. Actas das VIII Jornadas de Jovens em Investigação Arqueológica*, Colecção ArqueoArte, 4. Lisboa, 43-51 (2016).
41. Silva, F. & Vander Linden, M. Amplitude of travelling front as inferred from 14C predicts levels of genetic admixture among European early farmers. *Scientific Reports* **7**, 11985 (2017).
42. Goldberg, A., Mychajliw, A. M. & Hadly, E. A. Post-invasion demography of prehistoric humans in South America. *Nature* **532**, 232-235 (2016).
43. Brown, W. A. The past and future of growth rate estimation in demographic temporal frequency analysis: Biodemographic interpretability and the ascendance of dynamic growth models. *J. Archaeol. Sci.* **80**, 96-108 (2017).
44. Burnham, K. P. & Anderson, D. R. *Model Selection and Multimodel Inference: A Practical Information-Theoretic Approach* (2nd Ed). New York: Springer-Verlag (2002).
45. Burnham, K. P. & Anderson, D.R. Multimodel Inference: Understanding AIC and BIC in Model Selection. *Sociological Methods & Research* **33**, 261-304 (2004).
46. R Core Team (2017) *R: A Language and Environment for Statistical Computing. R Foundation for Statistical Computing*, Vienna, Austria. URL: https://www.R-project.org/

47. Mangado, X. (ed.) *El Paleolítico superior peninsular: novedades del siglo XXI. Homenaje al profesor Javier Fortea*. Monografías del SERP, 8. Barcelona (2010).
48. Sala, R. (ed.) Pleistocene and Holocene hunter-gatherers in Iberia and the Gibraltar Strait: The current archaeological record. Universidad de Burgos. Fundación Atapuerca (2014).
49. Rivas-Martínez, S. *et al.* Biogeografía de la península Ibérica, Islas Baleares y Canarias. *Folia Bot. Matritensis* **8**, 1-5 (1990).
50. Rasmussen, S.O. *et al*. A stratigraphic framework for abrupt climatic changes during the Last Glacial period based on three synchronized Greenland ice-core records: refining and extending the INTIMATE event stratigraphy. *Quat. Sci. Rev*. **106**, 14-28 (2014).
51. Martí, B., Aura, J.E., Juan Cabanilles, J., García, O. & Fernández, J. El Mesolítico geométrico de tipo "cocina en el País Valenciano. In *El Mesolítico Geométrico en la Península Ibérica [The Geometric Mesolithic in the Iberian Peninsula]* eds Utrilla P, Montes L*.,* Monografías Arqueológicas, 44, Zaragoza, 205-258 (2009).
52. Aura, J.E., Jordá, J.F., Montes, L. & Utrilla, P. Human responses to Younger Dryas in the Ebro valley and Mediterranean watershed (Eastern Spain). *Quat. Int.* **242**, 348-359 (2011).
53. Villaverde, V., Román, D., Pérez Ripoll, M. & Bergadà, M.M. The end of the Upper Paleolithic in the Mediterranean Basin of the Iberian Peninsula. *Quat. Int.* **272-273**, 17-32 (2012).
54. Straus, L.G. Environmental and cultural changes across the Pleistocene-Holocene transition in Cantabrian Spain. *Quat. Int.* **465**, 222-233 (2018).
55. Bicho, N. & Haws, J. The Magdalenian in central and southern Portugal: Human ecology at the end of the Pleistocene. *Quat. Int.* **272-273**, 6-16 (2012).
56. Utrilla, P. *et al.* The Ebro Basin in NE Spain: A crossroads during the Magdalenian. *Quat. Int.*, **272-273**, 88-104 (2012).
57. Soto, A. *et al.* Epipalaeolithic assemblages in the Western Ebro Basin (Spain): The difficult identification of cultural entities. *Quat. Int.* **364**, 144-152(2015).
58. Straus, L.G., González Morales, M., Fano Martínez, M.A. & García-Gelabert, M.P. Last Glacial Human Settlement in Eastern Cantabria (Northern Spain), *J. Archaeol. Sci.* **29**, 1403-1414 (2002).
59. Straus, L.G. & González Morales, M.R. The Magdalenian settlement of the Cantabrian region (Northern Spain): The view from El Miron Cave. *Quat. Int.*, **272-273**, 111-124 (2012).
60. Straus, L.G., González Morales, M., Higham, T., Richards, M. & Talamo, S. Radiocarbon dating the Late Upper Palaeolithic of Cantabrian Spain: El Mirón Cave date list IV. *Radiocarbon* **57**, 183-188 (2015).
61. Arias, P. & Ontañón, R. La Garma (Spain): Long-Term Human Activity in a Karst System. In Bergsvik, K.A. & Skeates, R. *Caves in Context. The Cultural Significance of Caves and Rockshelters in Europe*. Oxbow Books, 101-117 (2012).

62. Fontes, L.M., Straus, L.G. & González Morales, M.R. Lithic and osseous artifacts from the Lower Magdalenian human burial deposit in El Mirón cave, Cantabria, Spain. *J. Archaeol. Sci.* **60**, 99-111 (2015).
63. Fano, M.A. Un nuevo tiempo: El Mesolítico en la región Cantábrica. *Kobie* **8**, 337-402 (2004).
64. Fano, M.A., Cubas, M. and Wood, R. The first farmers in Cantabrian Spain: Contributions of numerical chronology to understand an historical process. *Quat. Int.* **364**, 153-161 (2015).
65. Mestres, J.S. & Arias, P. Datación por radiocarbono y calibración de las fechas radiocarbónicas aplicadas a materiales de origen terrestre y marino procedentes de la región cantábrica. In Clemente I. (coord.) *Explotación de Recursos Litorales y Acuáticos en la Prehistoria*. Workshop, Barcelona 15-16 Abril 2005. Departament d'Arqueologia i Antropologia-Institució Milà i Fontanals CSIC Archaeology & Anthropology Working Papers, 2) (2006).
66. García-Martínez, I. *et al.* Paleoambiente y cambios culturales en los inicios del Holoceno: el abrigo de Artusia (Unzué, Navarra). *Trabajos de Arqueología Navarra* **26**, 7-98 (2014).
67. González-Sampériz, P. *et al.* Patterns of Human occupation during the Early Holocene in the Central Ebro Basin (NE Spain) in response to the 8.2 ka climatic event. *Quat. Res.* **71**, 121-132 (2009).
68. Alday, A. *et al.* The silence of the layers: Archaeological site visibility in the Pleistocene-Holocene transition at the Ebro Basin *Quat. Sci. Rev.* **184**, 65-106 (2018).
69. Utrilla, P., Montes, L., Mazo, C., Martínez-Bea, M. & Domingo, R. El Mesolítico Geométrico en Aragón. In Utrilla, P. & Montes, L. *(eds.) El Mesolítico Geométrico en la Península Ibérica [The Geometric Mesolithic in the Iberian Peninsula]* Monografías Arqueológicas, 44, Zaragoza, 119-176 (2009).
70. Utrilla, P. & Domingo, R. The Mesolithic transition in the Ebro valley. In Manen, C., Perrin, T. & Guilaine, J. (eds.) *The Neolithic Transition in the Mediterranean*. Errance –AEP, 327-353 (2014).
71. Cacho, I. *et al*. Variability of the western Mediterranean Sea surface temperature during the last 25,000 years and its connection with the Northern Hemisphere climatic changes. *Paleoceanography* **16,** 40-52 (2001).
72. Fletcher, W. J., Sanchez, M. F., Peyron, O. & Dormoy, I. Abrupt climate changes of the last deglaciation detected in a Western Mediterranean forest record. *Clim. Past.* **6**, 245-264 (2010).
73. Román, D. *et al.* La fin du Paléolithique dans la Catalogne méridionale ibérique revisitée: nouvelles réponses pour anciennes questions, *L'Anthropologie* **120**, 610-628 (2016).
74. Fullola, J.M. *et al.* The Magdalenian in Catalonia (northeast Iberia). *Quat. Int.* **272-273**, 55-74 (2012).

75. Vaquero, M. & García-Argüelles, P. Algunas reflexiones sobre la ausencia de Mesolítico geométrico en Cataluña. In Utrilla, P. & Montes, L. *(eds.) El Mesolítico Geométrico en la Península Ibérica [The Geometric Mesolithic in the Iberian Peninsula]* Monografías Arqueológicas, 44, Zaragoza, 191-204 (2009).
76. Morales, J.I. *et al.* Paleogeografia humana durante el Tardiglaciar y Holoceno inicial en el ámbito mediterráneo del NE Ibérico. *Cuaternario y Geomorfología* **26**, 11-28 (2012).
77. Monteiro-Rodrigues, S. The Prehistoric site of Prazo (Northern Portugal): Preliminary data on the 8th millennium cal BC occupation. In Bicho, N., Detry, C., Price, T.D. & Cunha, E. (eds.) *Muge 150th: The 150th Anniversary of the Discovery of Mesolithic Shellmiddens*, vol.2, Cambridge Scholar Publishing, 17-27 (2015).
78. Araújo, A.C. Chapter One: A few steps backwards... in search of the origins of the Late Mesolithic. In Bicho, N., Detry, C., Price, T.D. & Cunha, E. (eds.) *Muge 150th: The 150th Anniversary of the Discovery of Mesolithic Shellmiddens*, vol.2, Cambridge Scholar Publishing, 1-12 (2015).
79. Zilhão, J. The Neolithic Transition in Portugal and the Role of Demic Diffusion in the Spread of Agriculture across West Mediterranean Europe. In Ammerman, A.J. & Biagi, P. (eds.) *The Widening Harvest. The Neolithic Transition in Europe: Looking Back, Looking Forward*. Boston: Archaeological Institute of America, 207-223.
80. Aldeias, V. & Bicho, N. Embedded behavior: Human activities and the construction of the Mesolithic Shellmound of Cabeço da Amoreira, Muge, Portugal. *Geoarchaeology* **31**, 530-549 (2016).
81. Bicho, N., Cascalheria, J., Marreiros, J., Pereira, T. The 2008-2010 excavations of Cabeço da Amoreira, Muge, Portugal. Mesolithic Miscellany **21**, 3-13 (2011).
82. Valente, M.J., Dean, R. & Carvalho, A.F. Shell Middens in Western Algarve (Southern Portugal) during the Mesolithic and Early Neolithic: Functionality, subsistence, and material culture. In Roksandic, M., Souza, S.M., Eggers, S. & Burchell, M. (eds.) *The Cultural Dynamics of Shell-Matrix Sites*. University of New Mexico Press, 75-90 (2014).
83. Cacho, C. *et al.* Human landscapes of the Late Glacial Period in the interior of the Iberian Peninsula: La Peña de Estebanvela (Segovia, Spain). *Quat. Int.* **272-273**, 42-54 (2012).
84. De la Torre et al. Chronological and palaeoenvironmental context of human occupations at the Buendía rock shelter (Central Spain) during the late Upper Pleistocene in inland Iberia. *J. Quat. Sci*. **30**, 376-390 (2015).
